# Immunohistochemical field parcellation of the human hippocampus along its antero-posterior axis

**DOI:** 10.1007/s00429-023-02725-9

**Published:** 2024-01-05

**Authors:** Emilio González-Arnay, Isabel Pérez-Santos, Lorena Jiménez-Sánchez, Elena Cid, Beatriz Gal, Liset M. de la Prida, Carmen Cavada

**Affiliations:** 1 Department of Anatomy, Histology and Neuroscience, Universidad Autónoma de Madrid, Madrid, Spain; 2 Department of Basic Medical Science-Division of Human Anatomy, Universidad de La Laguna, Santa Cruz de Tenerife, Canary Islands Spain; 3 Centre for Clinical Brain Sciences, University of Edinburgh, Edinburgh, UK; 4 Instituto Cajal, CSIC, Madrid, Spain; 5 Universidad CEU-San Pablo, Madrid, Spain

**Keywords:** Hippocampus, Parcellation, Segmentation, Hippocampal head, Hippocampal body, Hippocampal tail, *Uncus*, Vertical hippocampus, Gyrus *fasciolaris*, Posterior hippocampus, Intralimbic gyrus, Andreas Retzius gyri, Band of Giacomini, *Fasciola cinerea*

## Abstract

The primate hippocampus includes the dentate gyrus, *cornu ammonis* (CA), and subiculum. CA is subdivided into four fields (CA1-CA3, plus CA3h/hilus of the dentate gyrus) with specific pyramidal cell morphology and connections. Work in non-human mammals has shown that hippocampal connectivity is precisely patterned both in the laminar and longitudinal axes. One of the main handicaps in the study of neuropathological semiology in the human hippocampus is the lack of clear laminar and longitudinal borders. The aim of this study was to explore a histochemical segmentation of the adult human hippocampus, integrating field (medio-lateral), laminar, and anteroposterior longitudinal patterning. We provide criteria for head-body-tail field and subfield parcellation of the human hippocampus based on immunodetection of Rabphilin3a (Rph3a), Purkinje-cell protein 4 (PCP4), Chromogranin A and Regulation of G protein signaling-14 (RGS-14). Notably, Rph3a and PCP4 allow to identify the border between CA3 and CA2, while Chromogranin A and RGS-14 give specific staining of CA2. We also provide novel histological data about the composition of human-specific regions of the anterior and posterior hippocampus. The data are given with stereotaxic coordinates along the longitudinal axis. This study provides novel insights for a detailed region-specific parcellation of the human hippocampus useful for human brain imaging and neuropathology.

**Supplementary Information:**

The online version contains supplementary material available at 10.1007/s00429-023-02725-9.

## Introduction

The human hippocampus is in the medial temporal lobe. The gross morphological and cytoarchitectural organization of the hippocampus is species specific, also within the primate order (Rosene and van Hoesen 1977; Barbas and Blatt 1995; Insausti and Amaral 2004; Ding and Van Hoesen 2015). Differences have been reported in convolutions, relative width of cell layers and dendritic tree organization (Seress 2007; Strange et al. 2014), as well as boundary definition between areas (Fernandez-Lamo et al. 2019; Bienkowski et al. 2021). These differences have a bearing, among other factors, on the differential development of learning, perception, and episodic memory (Zeidman and Maguire 2016; Murray et al. 2018). Traditionally considered the morphological substrate for the acquisition of declarative memory in humans (Scoville & Milner 1957; Corkin 1984; Annese et al. 2014), the hippocampus is also involved in visuospatial memory, emotional memory, resilience, working memory and emotional reactivity (Phelps 2004; Yang and Wang 2017; Li et al. 2022). This functional versatility relates to its position as a node in cortical connectivity (Moscovitch et al. 2016) with a constellation of connections ranging from the orbitofrontal cortex to the posterior parietal and temporal cortices, particularly the entorhinal cortex (Witter et al. 1989; Witter and Amaral 1991, 2021). These connections are field specific and follow laminar and longitudinal (i.e., anteroposterior) patterns (Witter et al. 1989; Witter and Amaral 1991, 2021; Poppenk et al. 2013; Strange et al. 2014; Qi et al. 2016; Dalton et al. 2019; Genon et al. 2021) that are functionally correlated (*e.g., *Li et al. 2022) but still not fully known in humans.

The human hippocampus is crucially involved in diverse neurological conditions. In Alzheimer’s disease, neurofibrillary tangles, β-amyloid deposits, synaptic loss, and synaptic abnormalities appear early and most densely in medial temporal lobe structures (Arnold et al. 1991; Braak and Braak 1991): the transentorhinal and entorhinal cortices (Arnold et al. 1991; Domínguez-Álvaro et al. 2019), *cornu ammonis* (CA), subiculum, and dentate gyrus (Scheff and Price 1998; 2003). In refractory epilepsy, structures within the temporal lobe, and the hippocampus in particular, have been identified as the trigger of seizures of variable severity and symptomatology (Blümcke 2009; Thom 2014). Hippocampal sclerosis is the main histopathological finding in 36.4% of refractory epilepsies (Blümcke et al. 2017), with the functional impact on the patient depending on the affected hippocampal region (Blümcke et al. 2017). However, the neuropathological patterns are poorly systematized, and the value of subfield involvement of the hippocampus in patient management is not yet clearly proven (Coras and Blümcke 2015).

The human hippocampus offers a special challenge for segmentation, mainly in its anterior and posterior ends. Morphologically, it differs from rodents and even from non-human primates. The human dorsal hippocampus is almost atrophic, whereas the anterior hippocampus is a complex, gyrified region with primate-specific connectomics (Rosene and van Hoesen 1987; Cavada et al. 2000; Genon et al. 2021). Both the anterior and posterior hippocampus are involved in hippocampal sclerosis (Thom et al. 2012). Interestingly, specific abnormalities in the anterior hippocampus have been found in non-treated schizophrenia (Kalmady et al. 2017).

Diverse models of in vivo segmentation of the human hippocampus have been set through medical imaging (Yushkevich et al. 2015; Wisse et al. 2017). Most models do not coincide (Wisse et al. 2017); besides, many of them either do not include the anterior and posterior hippocampal ends, and/or they have not been histologically verified (Yushkevich et al. 2015; Tian et al. 2020; DeKraker et al. 2020). Traditional histological parcellation of the human hippocampus has relied on myelin and Nissl stainings (Rosene and van Hoesen 1977; Duvernoy 2005; Yushkevich et al. 2015), with new data emerging from the use of molecular markers like NeuN, parvalbumin, NPNFP, calretinin (Ding and van Hoesen 2015) or neurotensin (Bienkowski et al. 2021). These studies provide poor information on the whole hippocampus, with field identification being typically based on mild immunostaining intensity differences between adjacent fields and subfields (Ding and van Hoesen 2015). While recent genetically defined maps are revealing a complex marker regionalization of the hippocampus (Bienkowski et al. 2018; 2021), some of the emerging approaches cannot be directly applied to the analysis of surgical specimens, which in most cases are fragmented, making the identification of specific fields difficult.

Considering the relevance of the hippocampus in human conditions as well as the limitations of current segmentations, we undertook the present study to provide an immunohistochemical parcellation of the human hippocampus based on a novel set of antibodies with consistent staining patterns across hippocampal fields and throughout the whole longitudinal axis of the hippocampus. We emphasize field and subfield characterization in the anterior (hippocampal head) and posterior hippocampus (hippocampal tail), as compared with the ‘canonical’ middle hippocampus (hippocampal body). This framework (head–body–tail) is not only compatible with the classical morphological view, but it has been assessed and confirmed using functional neuroimaging and computational methods (Tian et al. 2020; DeKraker et al. 2020; Genon et al. 2021). To ensure comparability between the present study and neuroimaging segmentation protocols (e.g., Wisse et al. 2017), two human brains (P1, P2) were sliced in the coronal stereotaxic plane, thus providing anteroposterior (AP) coordinates for each macro or microanatomical landmark proposed. For simplicity, the term regionalization will refer to macroscopic regions of the human hippocampus whereas segmentation will be applied to the distinction between CA1-CA3/CA3h fields (as well as subiculum), and parcellation will be reserved to subfields within each major hippocampal field.

The nomenclature used along this article is according to the thorough study by Ding and van Hoesen (2015).

## Materials and methods

### Human tissue

Revealing precise topographical patterns of protein expression requires gold standard human brain tissue (perfused cases P1, P2). Such tissue was obtained and processed using a stringent method: very short *post-mortem* lapses, transcarotid and transvertebrobasilar perfusion and stereotaxic slicing. After an immunohistochemical marker was identified as suitable in cases P1 and P2, it was subsequently tested in brain tissue treated with different fixation methods and *post-mortem* gaps to test if the markers worked in the  several conditions present in clinical pathology laboratories. Altogether, four sets of human samples were used in this study: a) Perfused brains with stereotaxic sectioning (labeled ‘P’: P1 and P2); b) Immersion fixed brains with no stereotaxic sectioning (labeled ‘I’: I1-I5); c) Immersion fixed-paraffin embedded brains (labeled ‘Pf’: Pf1-Pf4); and d) a single brain extracted from a donor subjected to whole-body trans-femoral perfusion (labeled ‘F: case F19) (further details in Supplementary Table 1).

Brains from two patients, aged 58 and 67 years old (P1, P2), with no known previous history of neurological or psychiatric disease, were obtained from the division of Pathology of the University Hospital “La Paz”, during necropsy study. The brains were extracted after *post-mortem* delays of less than 3.5 h, and were fixed by perfusion through the carotids and the vertebral or basilar arteries with 4% paraformaldehyde. During perfusion, the brains rested within a brain cast. The brains were sliced in 1 cm coronal blocks in the stereotaxic canonical plane by means of a custom-designed device using the Talairach and Tournoux (1988) criteria for alignment and determination of the AP plane as described in detail in García-Cabezas et al. (2023). Tissue blocks containing the hippocampus were dissected and post-fixed in 4% paraformaldehyde in phosphate-buffer (PB) solution for 24 h. Blocks were subsequently immersed in gradually ascending concentrations of sucrose in PB for cryoprotection, stored at –20 ºC in a 30% glycerin–30% ethylene glycol solution, and cut into 50 µm-thick sections with a freezing microtome before further processing.

The immersion fixed brains I1–I5 were obtained from the Neurological Tissue Bank of Navarra and their use was approved by the Ethics Committee of this institution. Brains were extracted and cut fresh (not-stereotaxically) into 0.5- to 1-cm-thick coronal slices, which were fixed by immersion in 4% paraformaldehyde at 4 °C for 4–5 days. The slices were immersed for several weeks in a cryoprotecting solution of 30% sucrose in 0.1 M PB and then cut frozen in the same way as described above. Additionally, slices from four paraffin-embedded brains (Pf1-Pf4) were used, all of them obtained from the Institut d’Investigació Biomédica de Bellvitge. Those brains had been immersion fixed for at least 24 h at 4 °C in 4% paraformaldehyde. After fixation and dissection of the medial temporal region, the tissue blocks were dehydrated, embedded in paraffin, and cut in 20 µm-thick sections.

Reference series of sections spaced apart 500 μm were stained for Nissl substance with cresyl violet and a modified acetylcholinesterase staining protocol (Geneser-Jensen and Blackstad 1971; Cavada et al. 1995).

Brain F19 (from a voluntary donor to the department of Basic Medical Sciences of the University of La Laguna) was obtained after full body 4% paraformaldehyde perfusion through one femoral artery and was used for macroscopic examination and dissection. Patients’ data and further technical details for brain processing applied to their brains are disclosed in Supplementary Table 1.

The use of human *post-mortem* brains for research has been approved by the Ethics Committees of University Hospital La Paz in Madrid (HULP. PI-169), of Universidad Autónoma de Madrid (CEI-41–857), and the University of La Laguna (CEIBA reg. 2021–3113).

### Antibodies

Antibodies against proteins that have been described to show distinct regional patterns of staining (through either immunohistochemistry, in situ hybridization or RT-qPCR) along the mammalian hippocampus were tested in slices from gold standard cases P1 and P2. Information about all primary antibodies tested in this study, including their dilutions, is detailed in Supplementary Table 2. Immunohistochemical staining along the hippocampal long axis was performed using the antibodies that gave optimal and consistent staining: PCP4, Rph3, ChrA and RGS-14.

Purkinje-cell protein 4 (PCP4) is a calmodulin regulator protein that controls ATP-induced  Ca^2+^ release (Wang et al. 2013) and is involved in neurite outgrowth and neurotransmitter release (Harashima et al. 2011). PCP4 is widely expressed in the non-human mammalian forebrain (Rowell et al. 2010; Renelt et al. 2014). A Sigma–Aldrich® unconjugated polyclonal anti-PCP4 antibody was used in this study, produced in rabbit against the following aminoacidic sequence of the human native protein: GAGATNGKDKTSGENDGQKKVQEEFDIDMDAPETERAAVAIQSQFRKFQKKK, which reacts both to human and mouse tissue. Macaque monkey cerebellar tissue was used as positive control (Ziai et al. 1988).

Rabphilin3a (Rph3a) is a vesicle-associated presynaptic protein involved in vesicle trafficking and exocytosis in neuroendocrine cells (Shirataki et al. 1993; Chung et al. 1995). It is found in synaptic vesicles (Shirataki et al. 1993) where it has calcium and phosphatidylinositol 4,5-bisphosphate binding properties. A Sigma–Aldrich® unconjugated polyclonal anti-Rph3a antibody was used in this study, produced in rabbit against the following aminoacidic sequence of the human native protein: SPAGLRRANSVQASRPAPGSVQSPAPPQPGQPGTPGGSRPGPGPAGRFPDQKPEVAPSDPGTTAPPREERTGGVGGYPAVGAREDRMSHPSGPYSQASAAAPQPAAARQPPPPEEEEEEANSYDSDEATTLGALEFSLLYDQDNS. Extensive neuropil reactivity in cortical grey matter and caudate tail was used as internal positive control.

Chromogranin A (ChrA) is the oldest known member of the granin protein family, acidic proteins that are a main component of secretory dense core vesicles from both the endocrine and neuroendocrine systems. Chromogranin A and chromogranin B are essential constituents of the intravesicular matrix where they act as  Ca^2+^ binders and aggregate at acidic pH (Yoo and Albanesi 1991; Videen et al. 1992). They are also essential to vesicle filling (Dominguez et al. 2018). ChrA in human hippocampus is present in CA2 pyramidal cells, granule cells and mossy fibers (Kandlhofer et al. 2000). A purified IgG monoclonal anti-chromogranin A antibody was used in this study (Wilson and Lloyd 1984; LK2H10, Cell Marque^®^). A paraffin section from a human neuroendocrine breast tumor was used as positive control.

Regulation of G protein signaling-14 (RGS-14) is a bridging molecule involved in downstream pathways arising from G protein-coupled receptors (Traver et al. 2000). It is present in rodent CA2 cells, where it plays a role in excitability and LTP at synapses in the *stratum radiatum,* associated to perineural nets (Zhao et al. 2007; Lee et al. 2010; Hayani et al. 2018). A Proteintech^®^ polyclonal IgG anti-RGS-14 antibody (16258–1-AP) was used in this study, produced against the following aminoacidic sequence of the human RGS-14:

KSLPLGVEELGQLPPVEGPGGRPLRKSFRRELGGTANAALRRESQGSLNSSASLDLGFLAFVSSKSESHRKSLGSTEGESESRPGKYCCVYLPDGTASLALARPGLTIRDMLAGICEKRGLSLPDIKVYLVGNEQALVLDQDCTVLADQEVRLENRITFELELTALERVVRISAKPTKRLQEALQPILEKHGLSPLEVVLHRPGEKQPLDLGKLVSSVAAQRLVLDTLPGVKISKARDKSPCRSQGCPPRTQDKATHPPPASPSSLVKVPSSATGKRQTCDIEGLVELLNRVQSSGAHDQRGLLRKEDLVLPEFLQLPAQGPSSEETPPQTKSAAQPIGGSLNSTTDSAL.

### Immunohistochemistry

Selected slices from the P and I brains were retrieved from the cryoprotection solution at least 12 h before free-floating immunohistochemistry. After clearing the cryoprotection solution in Tris buffer (TB) 0.1 M pH7.6, endogenous peroxidase inactivation was performed by immersion in a 10% methanol + 3% H_2_O_2_ in bidistilled H_2_O (ddH_2_O) for 30 min. After further rinsing, antigen retrieval was performed for one hour in a preheated water bath at 90ºC, with the slices submerged in a buffered acid solution (38 ml citric acid 0.1 M + 166 ml sodium citrate 0.1 M + ddH_2_O up to 400 ml; adjusted with glacial acetic acid to pH6). A 3 h-long preincubation in a TB saline (TBS)-Normal Goat Serum (NGS) 4%-Triton X-100 0.2% solution followed. Incubation in the primary antibody was performed at 4ºC during 60 h using the following dilutions in TBS-NGS 4%-Triton X-100 0.2%: 1/750 for PCP4 (rabbit polyclonal, Sigma–Aldrich^®^ HPA005792), 1/500 for Rph3a (rabbit polyclonal, Sigma-Aldrich^®^ HPA002475), 1/100 for ChrA (mouse monoclonal, Cell Marque^®^ LK2H10), and 1/150 for RGS-14 (rabbit polyclonal, Proteintech^®^ 16258–1-AP). Incubation in secondary antibody was performed during two hours at room temperature after rinsing in TBS (goat anti-rabbit, Millipore^®^, AP187B or goat anti-mouse, Jackson, 115.065.003; 1/500; both in TBS-NGS 4%-Triton X-100 0.2%). The sections were rinsed in TBS and then incubated in ABC Vectastain^®^ solution at room temperature for 2 h. After consecutive rinses in TBS/TB for 15 min, immunoreaction was performed using cold H_2_O_2_ 0.003% + 0.05% DAB in TB. Sections were mounted in gelatinized slides, dehydrated, defatted, and covered with DePeX^®^. When surface immunohistochemistry was performed on Pf brain slices directly over slides, the protocol was similar, albeit using adequate volumes and omitting Triton X-100.

### Immunofluorescence

Immunofluorescence was performed in tissue from the I brains. Antigen retrieval and primary antibody incubation were performed as described above. In addition to the above-mentioned antibodies, an anti-parvalbumin primary antibody was used in immunofluorescence experiments (mouse monoclonal, Swant PV235, or rabbit polyclonal, Swant PV27; 1/4000). Before antigen retrieval, sections were rinsed for 20 min in 50 nM NH_4_Cl to decrease background staining. Incubation in conjugated secondary antibody was performed during two hours at room temperature in a light-protected environment (goat anti-rabbit Alexa Fluor 568 Invitrogen^®^ R371171/100, and goat anti-mouse Alexa Fluor 488 Invitrogen^®^ R37120 1/100). Incubation in Bisbenzimide H33258 was performed for 10 min (Sigma–Aldrich^®^ B2883; 1/10000) for anatomical reference. Free-floating sections were mounted and coverslipped with DePeX^®^ or Mowiol (17% polyvinyl alcohol 4–88, 33% glycerin and 2% thimerosal).

### Image analysis

For DAB-immunostained sections, low power mosaic images of every stained section were obtained with a 1.25 × objective in a Zeiss Axioskop microscope equipped with a digital camera and processed with Stereo Investigator^®^ software (v9, MicroBrightField^®,^ USA). Higher magnification pictures of specific tissue details were taken with 10 × and 20 × objectives. For immunofluorescence sections, image analysis was performed using confocal microscope fluorescence stacks obtained from superficial imaging planes (Leica SP5; v2.6.0LAS AF software).

## Results

**Fig. 1:**
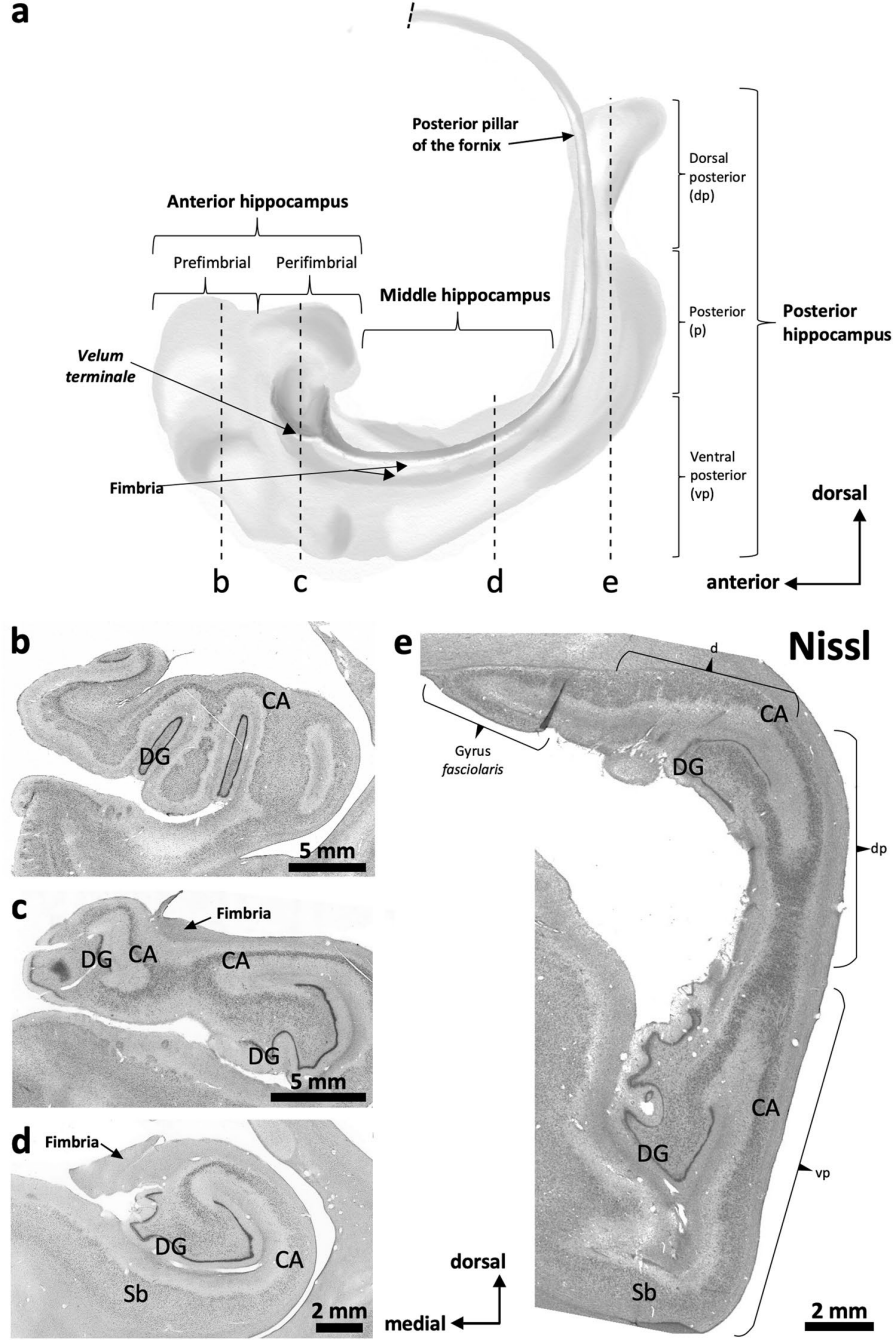
Macroscopic regionalization of the human hippocampus (**a**). There are three main regions from anterior to posterior. The most anterior one, anterior hippocampus or hippocampal head (**b** and **c**), bends back on itself surrounding anteriorly the anterior end of the fimbria defining a prefimbrial (**b**) and a perifimbrial region (**c**) that is continued posteriorly with the middle hippocampus or hippocampal body (**d**). Once the fimbria acquires a vertical orientation and becomes the posterior pillar or *crus* of the fornix, a C-shaped concave posterior hippocampus appears (**e**). This posterior hippocampus is divided into a ventral posterior region (vp; ventral posterior hippocampus), a dorsal posterior region (dp, dorsal posterior hippocampus), and the posterior region (p, posterior hippocampus ‘proper’) which forms the posterior end of the hippocampus. A dorsal hippocampal region (d, dorsal hippocampus) links the dorsal posterior hippocampus and the posterior hippocampus proper –not shown in **e**– with the gyrus *fasciolaris* (**e**), which lies ventral to the posterior end of the corpus callosum. Abbreviations: CA, *cornu ammonis;* DG, dentate gyrus; Sb, subiculum

Below we describe, first, the features of the middle ‘canonical’ hippocampus (hippocampal body), which is equivalent across species and whose fields are easy to identify (Fig. 1d); next, we describe the anterior hippocampus (hippocampal head) and the posterior hippocampus (hippocampal tail), including the non-canonical hippocampal regions, such as those in the *uncus* and the gyrus *fasciolaris*. Figure 1 offers a graphical summary of the regions analyzed. Supplementary Tables 3–9 provide summaries of the main immunohistochemical features, as well as information on coordinates and microanatomical landmarks, of the hippocampal regions described below.

We use the topographical terms medial and lateral (relative to the midline) to describe the human hippocampal fields instead of the terms proximal and distal (relative to the dentate gyrus) often used in the rodent literature. The convoluted shapes of the human hippocampus in its anterior and posterior ends justify the choice of the medial/lateral topographical nomenclature.

### The human middle hippocampus

#### Protein immunodetection in the human hippocampus

Four of the fourteen proteins tested (PCP4, Rph3a, ChrA, RGS-14) showed staining patterns that allowed consistent parcellation of the human hippocampus. The remaining ten produced staining patterns that were either negative (Astn2, Dlk1), not consistent between experiments (Coch1) or produced staining patterns that did not allow reliable field parcellation, layer identification or longitudinal regionalization (Nr3c2, Col11A1, Neurotensin, Amigo1, Dclk3, Itga7, Wfs-1). Further information on the negative and inconclusive findings in humans is summarized in Supplementary Table 2; this table additionally includes data on expression patterns of the tested antibodies, or of their corresponding proteins, in the murine hippocampus.

#### PCP4, Rph3a, ChrA and RGS-14 show distinct patterns of expression along the hippocampal medio-lateral (field) axis

**Fig. 2:**
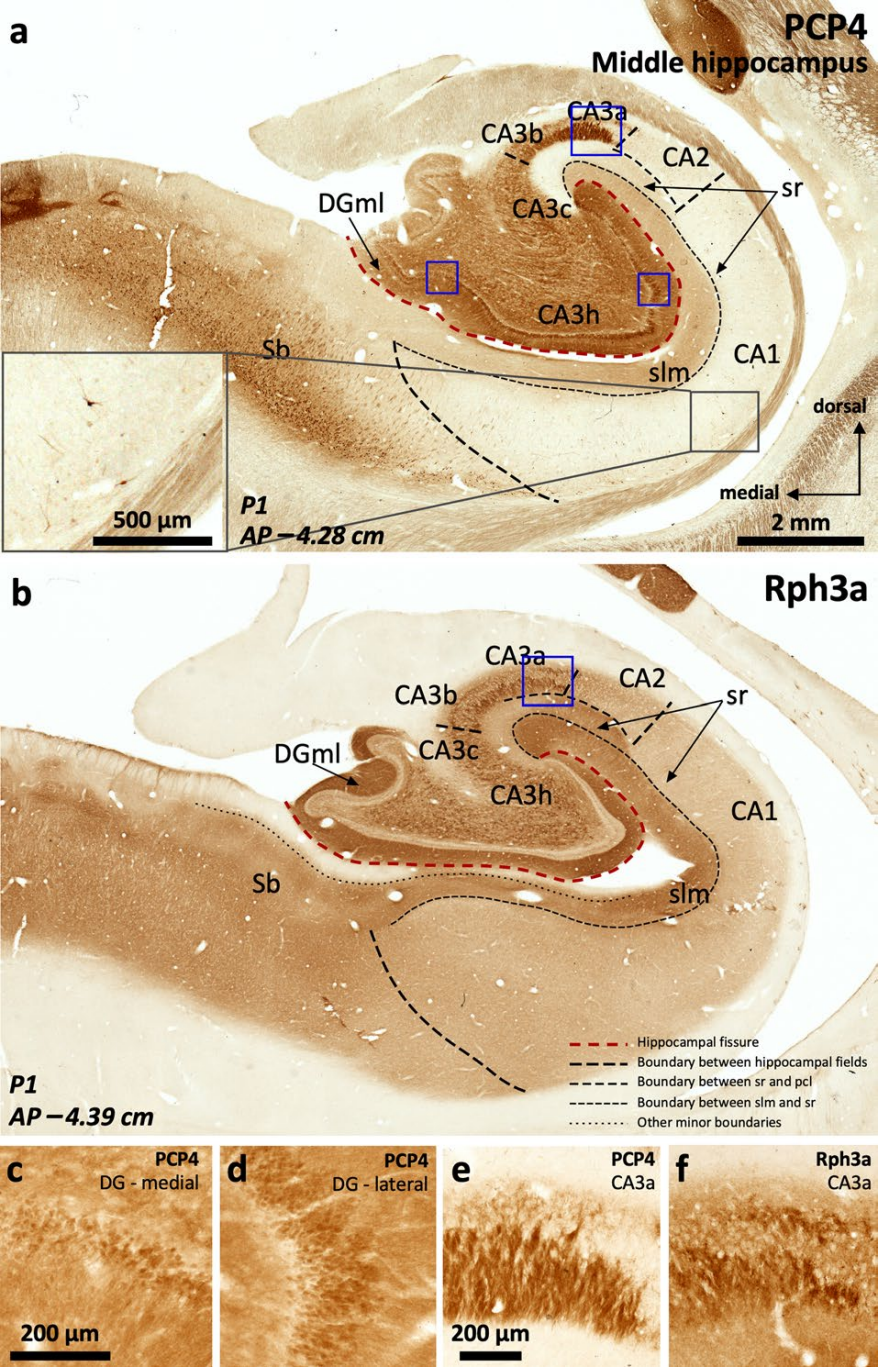
Distribution of immunohistochemical markers PCP4 (**a**) and Rph3a (**b**) in the human middle hippocampus. Blue squares in (**a**) and (**b**) identify fields enlarged in **c**–**f**. The dentate gyrus molecular layer (DGml) shows neuropil immunoreactivity for PCP4 and Rph3a. In the hilar region (CA3h), Rph3a is present in neuropil and interneurons (b, see also Supplementary Fig. 1). A dense plexus of PCP4 + , Rph3a + mossy fibers reaches lateral CA3 (CA3a, CA3b) thus highlighting the medial boundary of CA2. The boundary between CA2 and CA1 is defined by a PCP4 + , Rph3a + *stratum radiatum* (sr) in CA2, in contrast to the PCP4–, Rph3a– *stratum radiatum* (sr) of CA1. Meanwhile, the boundary between CA1 and subiculum is defined by the lack of immunostaining of PCP4 in pyramidal cells of CA1 in contrast to PCP4 + pyramidal cells in subiculum; The inset in (**a**) shows some scattered non-pyramidal neurons in the *stratum oriens* of CA1. **c, d,** Enlarged regions from the dentate gyrus -DG- in (**a**) showing cell somata immunoreactive for PCP4 in the granule cell layer. Note the less marked staining of cell somata in the medial DG (**c**) compared to the lateral DG (**d**). **e, f,** high magnification (from **a** and **b**, respectively) of the PCP4 + (**e**), Rph3a + (**f**) mossy fibers reaching lateral CA3

PCP4 immunoreactivity (Fig. 2a) is present in the dentate gyrus (Supplementary Table 3), CA, and subiculum (Supplementary Table 4). In the dentate gyrus, PCP4 stains the somata of the granule cell layer, slightly less marked medially (Fig. 2a, c, d), as well as the neuropil of the molecular layer and the hilus. In CA3, PCP4 stains the neuropil of all layers, but particularly deep and superficial mossy fibers (Fig. 2a, e). In the human CA2 there is light neuropil PCP4 immunoreactivity (PCP4 +) in the *stratum lacunosum moleculare* as well as a subtle but consistent neuropil staining in the *stratum radiatum;* no PCP4 immunoreactivity was present in CA2 cell bodies (Fig. 2a). Actually, contrary to what is described for rodents (Renelt et al. 2014), CA pyramidal cells are PCP4 negative (PCP4–) (Fig. 2a, e). In CA1, PCP4 immunostaining is limited to the superficial *stratum lacunosum moleculare* and some scattered non-pyramidal neurons in the *stratum oriens* (inset in Fig. 2a). The subiculum shows the clearest pattern of PCP4 immunostaining, where it highlights the somata and dendritic trees of the pyramidal cells (Fig. 2a).

Rph3a (Fig. 2b) is expressed in the dentate gyrus (Supplementary Table 3), CA and subiculum (Supplementary Table 4). In the dentate gyrus, Rph3a strongly highlights the outer molecular layer (Fig. 2b, Supplementary Table 3), while granule cells and the subgranular zone are Rph3a negative. In the hilar region, Rph3a is diffusely present in neuropil and in both parvalbumin + and parvalbumin– interneurons (Supplementary Fig. 1). Parvalbumin+ neurons are concentrated in the subgranular zone while parvalbumin– neurons are scattered in deeper hilar regions. In CA, Rph3a stains neuropil across all the layers; its expression matches the expression of PCP4 in the deep and superficial mossy fibers of CA3 (Fig. 2a, b, e, f). In CA2, there is moderate Rph3a staining in *strata radiatum* and *lacunosum moleculare.* The most superficial part of *stratum lacunosum moleculare* of medial CA1 regions next to the subiculum is almost devoid of Rph3a staining (Fig. 2b). In the subiculum, the superficial part of the *stratum lacunosum* lacks Rph3a immunoreactivity. The whole depth of the pyramidal layer of CA and the subiculum shows also diffuse Rph3a neuropil staining (Fig. 2b, Supplementary Table 4).

**Fig. 3:**
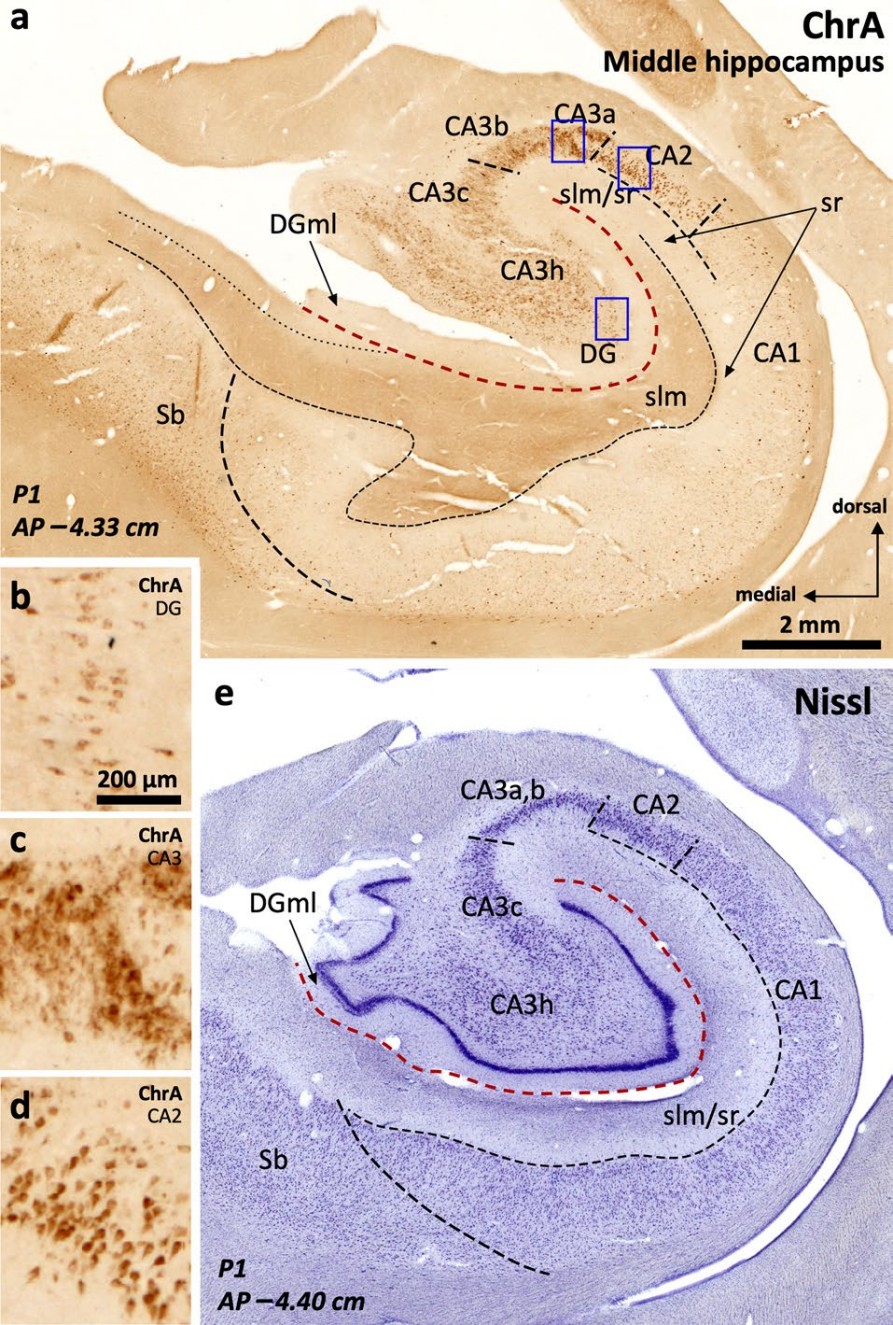
Parcellation of the human middle hippocampus using ChrA immunohistochemistry (**a**) and Nissl staining (**e**). Blue squares in (**a**) identify fields enlarged in **b–d**. ChrA immunostaining shows immunoreactive somata in the granule cell layer of the dentate gyrus (DG) (**a**, high magnification in **b**). ChrA immunostaining reveals boundaries between CA fields: CA3 presents mostly neuropil staining, together with some ChrA somata (**c**), CA2 presents pyramidal cells showing ChrA + cytoplasmic staining (**d)**, and CA1 shows ChrA + neuropil in s*tratum lacunosum moleculare* (slm), ChrA– neuropil in *stratum radiatum* (sr), and ChrA + cytoplasmic staining in some scattered non-pyramidal cells in the deep pyramidal layer and *stratum oriens*; a similar pattern is present in the subiculum (Sb). **e,** Nissl staining of a histological section in a close anteroposterior level to that of (a). Abbreviation: DGml, dentate gyrus molecular layer. The code for the broken lines as in Fig. 2b

ChrA immunostaining (Fig. 3a and Supplementary Fig. 1) is mostly limited to CA and the subiculum (Supplementary Tables 3 and 4). In the dentate gyrus, some neuronal bodies are ChrA + in the subgranular layer (Fig. 3a, b). In CA, ChrA is expressed in the neuropil of CA3, as well as in scattered neurons across CA fields. In CA3, diffuse, strongly labeled, ChrA + terminals are present mostly laterally, overlapping with PCP4 + , Rph3a + mossy fibers (Fig. 3a, c). ChrA + neurons in CA3 and CA2 show strong punctate perikaryal staining in the somata (Fig. 3c, d); ChrA + cell bodies in CA2 are pyramidal neurons (Fig. 3a, d). Both features, neuropil and neuronal ChrA + staining, allow to draw a medial boundary for CA2 with better precision than cytoarchitecture (see comparison between Fig. 3a and 3e). In CA1, ChrA shows subtle neuropil immunostaining in deep *stratum lacunosum moleculare* along with some scattered non-pyramidal cells in deep pyramidal layer and *stratum oriens*, a similar pattern is also present in subiculum (Fig. 3a, Supplementary Table 4).

**Fig. 4:**
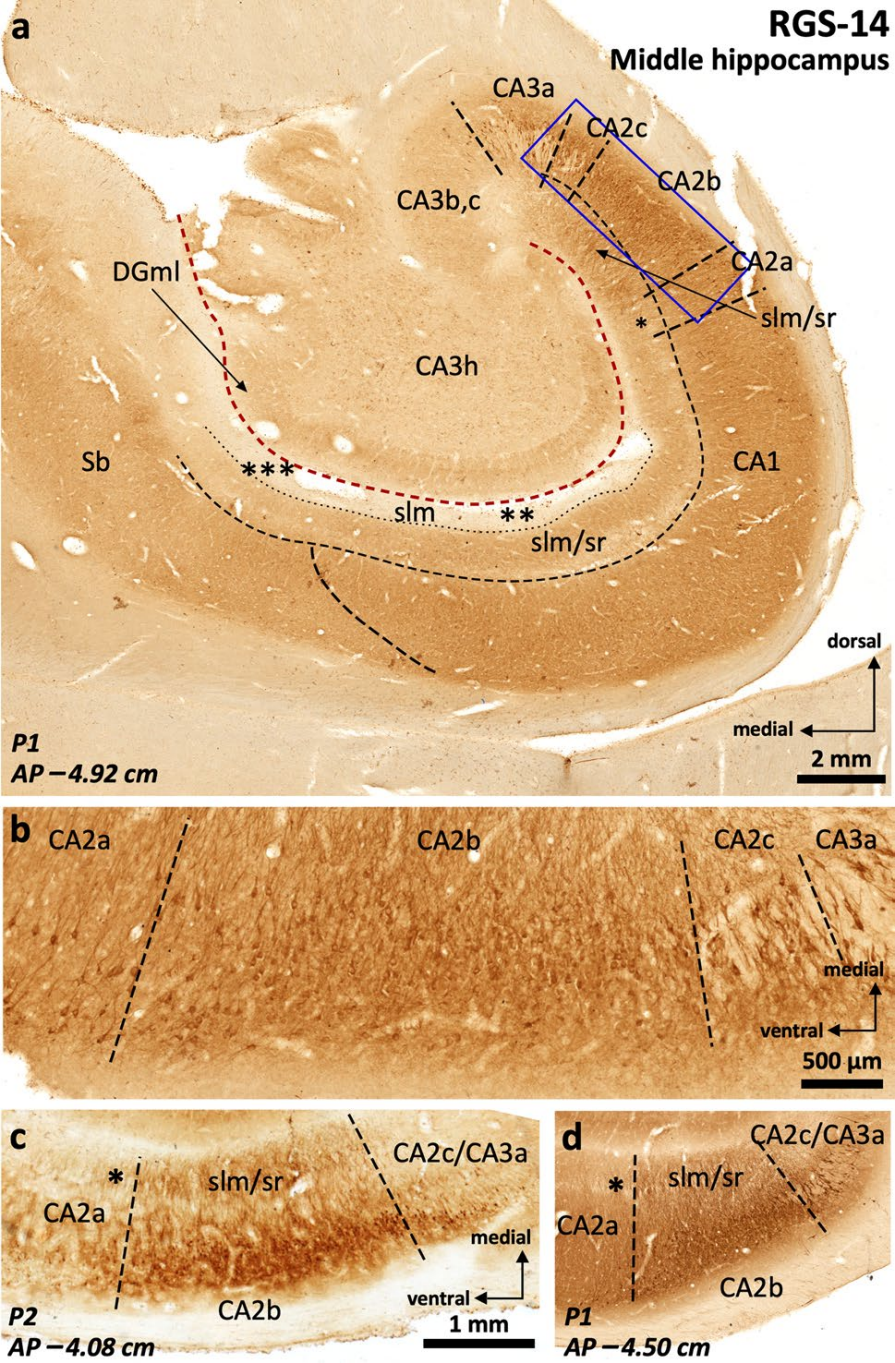
RGS-14 immunostaining in human middle hippocampus (**a**) and RGS-14-based compartmentalization of field CA2 (**b**-**d**). The blue square in (**a**) refers to the field shown at higher magnification in (**b**). RGS-14 is diffusely and sparsely present in hilar neuropil (h), as well as throughout the whole depth of CA3b, CA3c (CA3b,c), and CA3h pyramidal cell layer. The presence of deep RGS-14 + pyramidal cells allows the demarcation of CA3a. The main medio-lateral dimension of CA2 (CA2b) features an intense RGS-14 + pyramidal cell layer, RGS-14 + dendrites are also found in the *stratum radiatum* (sr) and *in stratum lacunosum moleculare* (slm). Note that an internal boundary between both *strata* is not distinguishable with this staining. A subset of RGS-14 + pyramidal cells extends further laterally, progressively intermingling with deep CA1 pyramidal cells and progressively losing the sr/slm immunoreactivity (* in **a**, **c**, **d**), this short subfield is termed CA2a. RGS-14 neuropil immunoreactivity in the slm tends to disappear in ventromedial regions of CA1 (** in **a**) and is not present in subicular slm (*** in **a**). Note the inter-individual differences in RGS-14 neuropil staining (**b**–**d**). Abbreviation: DGml, dentate gyrus molecular layer. The code for the broken lines as in Fig. 2b

RGS-14 immunoreactivity (Fig. 4) is present in the dentate gyrus, CA, and subiculum (Supplementary Tables 3 and 4). In the dentate gyrus, RGS-14 stains nonspecifically the neuropil. In CA, neuropil staining is present in all layers except in the most superficial half of ventro-medial CA1 *stratum lacunosum moleculare* (Fig. 4a). The main feature of RGS-14 immunoreactivity in CA is its presence in the somata and apical dendrites of pyramidal neurons of CA2 and lateral CA3 (Fig. 4b-d); in CA2, RGS-14 colocalizes with ChrA (Supplementary Fig. 1d-f). RGS-14 also shows progressively fainter lateral to medial neuropil staining in CA1 and subiculum, being also helpful to set a boundary between CA2 and CA1 (see below; Fig. 4, Supplementary Table 4). Immunohistochemical features of RGS-14 in the subiculum are similar as those in medial CA1.

#### PCP4 and Rph3a are useful as immunohistochemical markers for field delimitation

The strong PCP4 and Rph3a immunohistochemical staining of mossy fibers reaching CA3 allows a precise subdivision of CA3 (Fig. 2). Notably, this is in contrast to rodents where the mossy fibers reach and establish synaptic contacts with the proximal aspect of the CA2 region (Fernandez-Lamo et al. 2019, see Discussion). The CA1/subiculum border is marked by the appearance of PCP4 + pyramidal neurons in subiculum (Fig. 2a). Deep scattered non-pyramidal PCP4 + cells are more evident in the *stratum oriens* of ventro-medial CA1 as compared to its dorso-lateral aspect (see Supplementary Table 4 and Fig. 2a, inset). All the staining patterns and boundaries here described are constant and independent of *post-mortem* delays and fixation method, albeit in paraffin embedded sections Rph3a shows the best results using a standard protocol.

#### Specific immunostaining patterns allow the identification of CA2

The strong PCP4 and Rph3a immunoreactivity present in mossy fibers and the *stratum radiatum* allow a clearcut delineation of the boundary between CA3 and CA2 (Fig. 2a, b). ChrA immunoreactivity is evident in pyramidal cells of CA2 as compared to CA3, where ChrA immunostaining is mostly present in the neuropil (Fig. 3a, c, d). The delimitation of these fields using ChrA immunostaining is coherent with the delimitation of the same fields using either PCP4 or Rph3a (Fig. 2). Therefore, CA2 borders can be set using any of the above three markers. RGS-14 stains CA2 pyramidal neurons and, in addition, serves to identify subfields within CA2 (Fig. 4, Supplementary Table 4 and Supplementary Fig. 1).

#### RGS-14 defines three discrete territories in human CA2

The most outstanding feature of RGS-14 immunostaining is its strong, constant, immunoreactivity in CA2 pyramidal cells (Fig. 4b-d). This immunoreactivity is organized into three discrete zones: CA2c (at the border with CA3), CA2b, and CA2a (a narrow transitional area at the border with CA1). In CA2a and CA2c, only deep pyramidal cells are RGS-14 + , while in CA2b, RGS-14 + neurons are present in the full depth of the pyramidal layer (Fig. 4b). The medial border of CA2 (CA2c) overlaps with lateral CA3 (CA3a). This overlapping region shows PCP4 + , Rph3a + immunostained mossy fibers (Fig. 2e, f) coextensive with RGS-14 immunostaining in deep pyramidal cells (Fig. 4b-d). Also, dense RGS-14 immunostaining in the neuropil is present in *stratum radiatum/stratum lacunosum moleculare* of CA2b, while this immunostaining becomes fainter towards CA2a and CA2c (Fig. 4a, c, d). Interestingly, this mediolateral organization of CA2 has echoes across species. We deal with this issue in the Discussion.

### The human anterior hippocampus

#### Macroscopic regionalization of the human anterior hippocampus

**Fig. 5:**
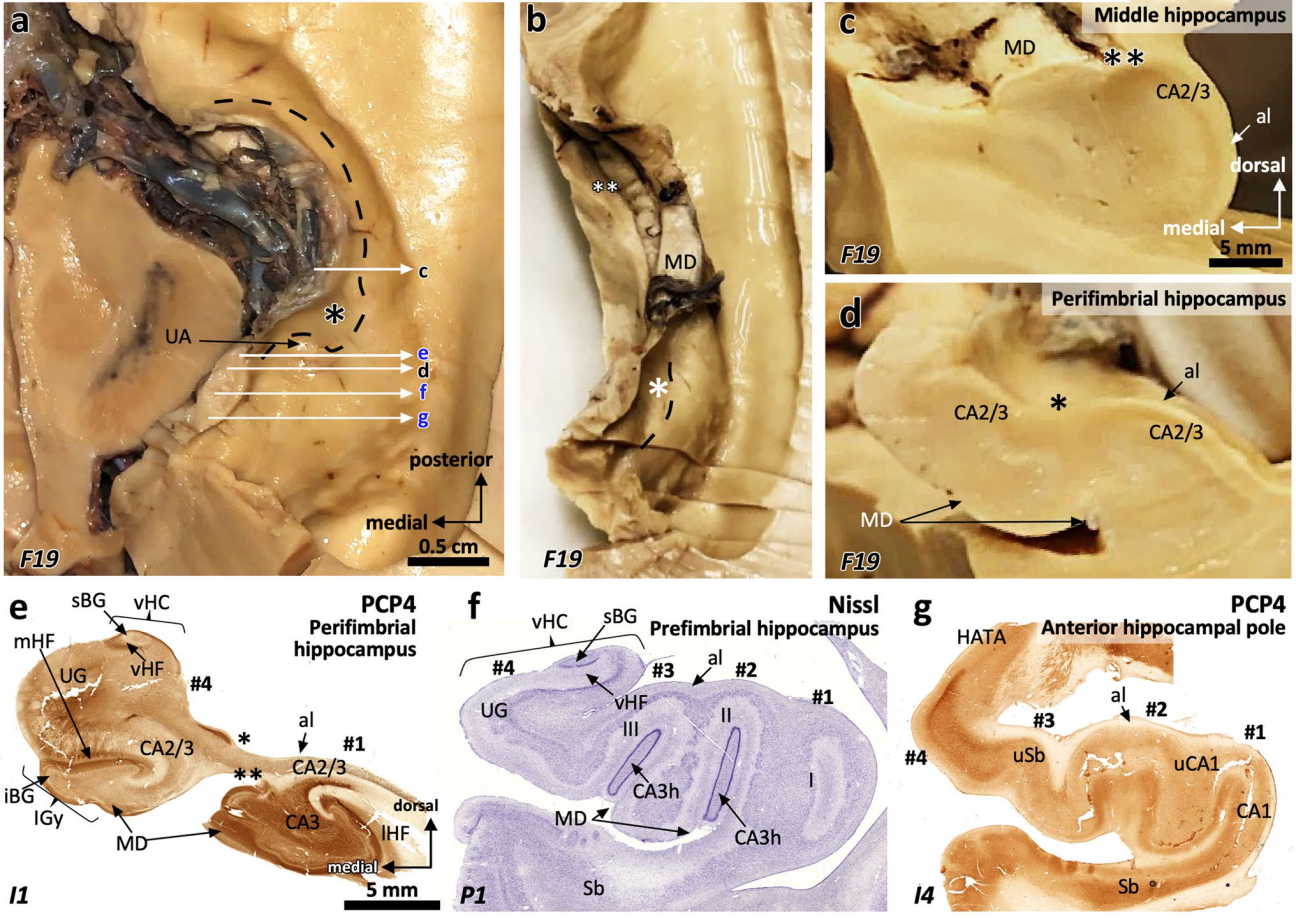
Macro (**a**-**d**) microscopic (**e**–**g**) correlation in the human anterior hippocampus (hippocampal head). Dorsal view of a left human hippocampus (**a**, prior to dissection, **b**, partially dissected). White arrows in (**a**) depict approximate levels of cutting to obtain the macroscopic (black characters) and microscopic (blue characters) sections shown in this figure. The hippocampal head is organized around the anterior end of the fimbria (* in **a**, **b**, **d**, **e**). Therefore, any region showing a macroscopic recognizable medial fimbriodentate junction (** in **b**,**c**) should be considered as middle hippocampus or hippocampal body. Note that the fimbriodentate junction is also present in **e** (**), but it is not easily recognizable macroscopically. Anterior to the middle hippocampus, three gross anatomical regions are present: **1.** Perifimbrial hippocampus (**e**) including the most lateral (#1) and medial (#4) hippocampal digitations, separated by the anterior fimbria (*) which is the main gross feature of this region along with the presence of a *margo denticulatus* (MD) laying ventrally (**d**, **e**) and not medially (**c**) to the fimbriodentate junction (**). **2.** Prefimbrial hippocampus (**f**): There is no fimbriodentate junction, but a minute *margo denticulatus* (MD, the surface of the dentate gyrus) is still present in the ventral surface of the hippocampal head. All classical hippocampal fields are represented. Intermediate hippocampal digitations (#2, #3) are present, including their dentate gyrus (II, III). **3.** Anterior hippocampal pole (**g**), which is the most anterior area, and where only subicular modified subfields and CA1 fields are present (uSb, uCA1). Abbreviations: al, *alveus*; -h as a suffix, hilar; HATA, hippocampo-amygdaloid transitional area; iBG, inferior Band of Giacomini; IGy, intralimbic gyrus; lHF, lateral hippocampal fissure; MD, *margo denticulatus*; mHF, medial hippocampal fissure; sBG, superior Band of Giacomini; Sb, subiculum; u- as a prefix, uncal; UA, uncal apex; UG, uncinate gyrus; vHC, vertical hippocampus; vHF vertical hippocampal fissure

**Fig. 6:**
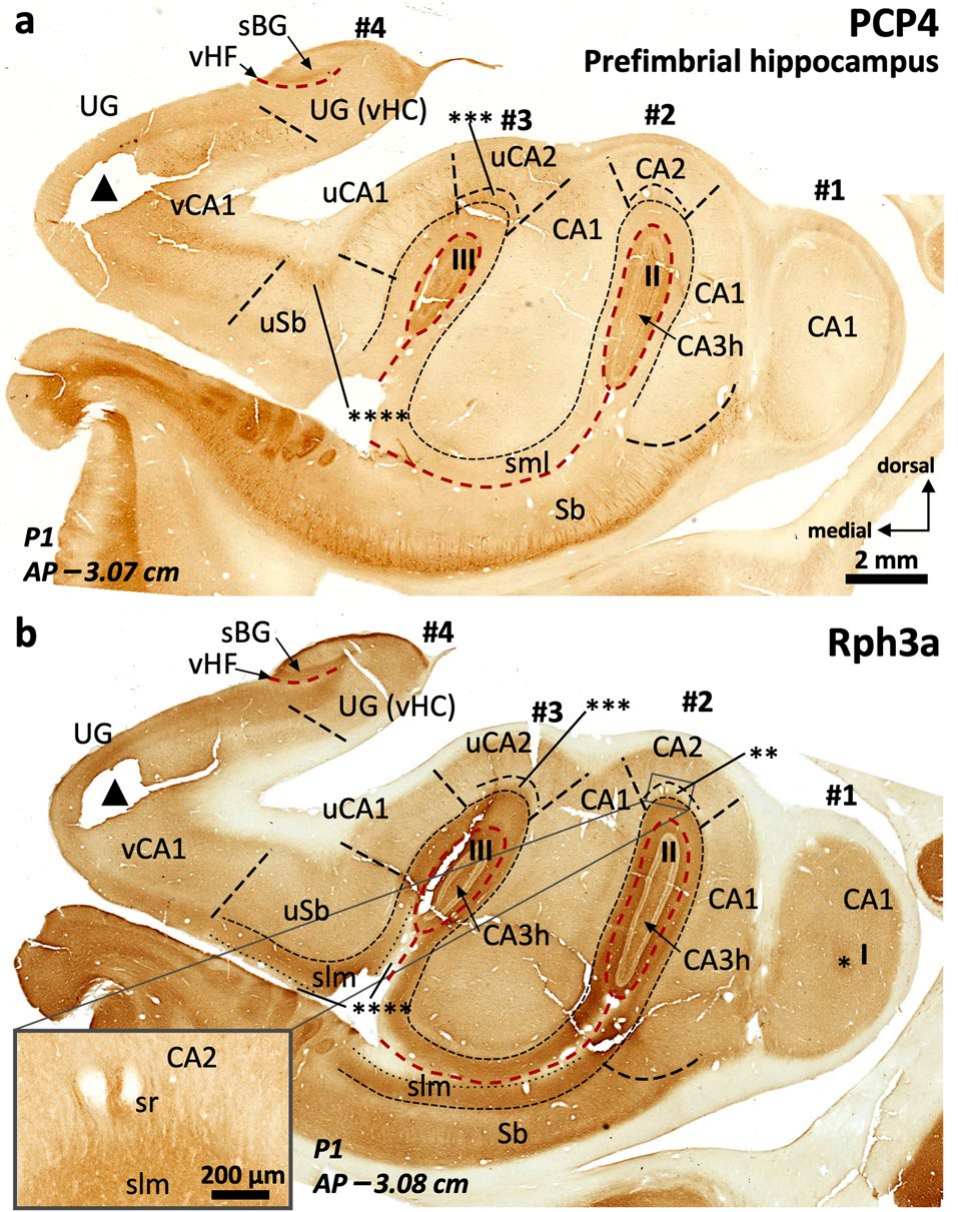
Field delimitation in the human prefimbrial hippocampus. Fields in the lateral digitations (#1, #2) follow a middle hippocampus-like pattern. Level –3,08 (**b**) is remarkable as it shows the most anterior histological evidence of field CA2 (a Rph3a + *stratum radiatum* (sr) ** in **b**, inset in **b**) and the most anterior *stratum lacunosum moleculare* (slm) of CA1 corresponding to digitation #1. Single * in **b** is at the most anterior end of digitation #1 dentate gyrus. Fields in the medial digitations (#3, #4) are strongly modified. Uncal CA1 (uCA1) shows a deep, discontinuous PCP4 + pyramidal cell layer (**a**) with strongly positive ascending projections. This deep PCP4 + layer is also present in uncal CA2 (uCA2), which still shows a lightly Rph3a + , PCP4 + *stratum radiatum* (*** in **a**, **b**). The uncal subiculum (uSb) shows rather typical features presenting both PCP4 immunoreactive cells at the pyramidal cell layer (**a**) and weaker Rph3a neuropil staining in the superficial part of slm (****) as compared to deeper part of this *stratum* (**b**, compare with Fig. 2). Note the density of PCP4 + cells (**a**) in the vertical CA1 (vCA1) deep pyramidal layer and how it narrows and bends dorsally upon converging with the remaining vertical hippocampus proper (vHC), in the uncinate gyrus (UG), where a vertical hippocampal fissure (vHF) is also identifiable. Triangles are within a tissue loss zone. Abbreviations: -h as a suffix; hilar; sBG, superior Band of Giacomini; Sb, subiculum; u- as a prefix, uncal; I, II, III, dentate gyrus of digitations #1, #2, #3, respectively. The code for the broken lines as in Fig. 2b

**Fig. 7:**
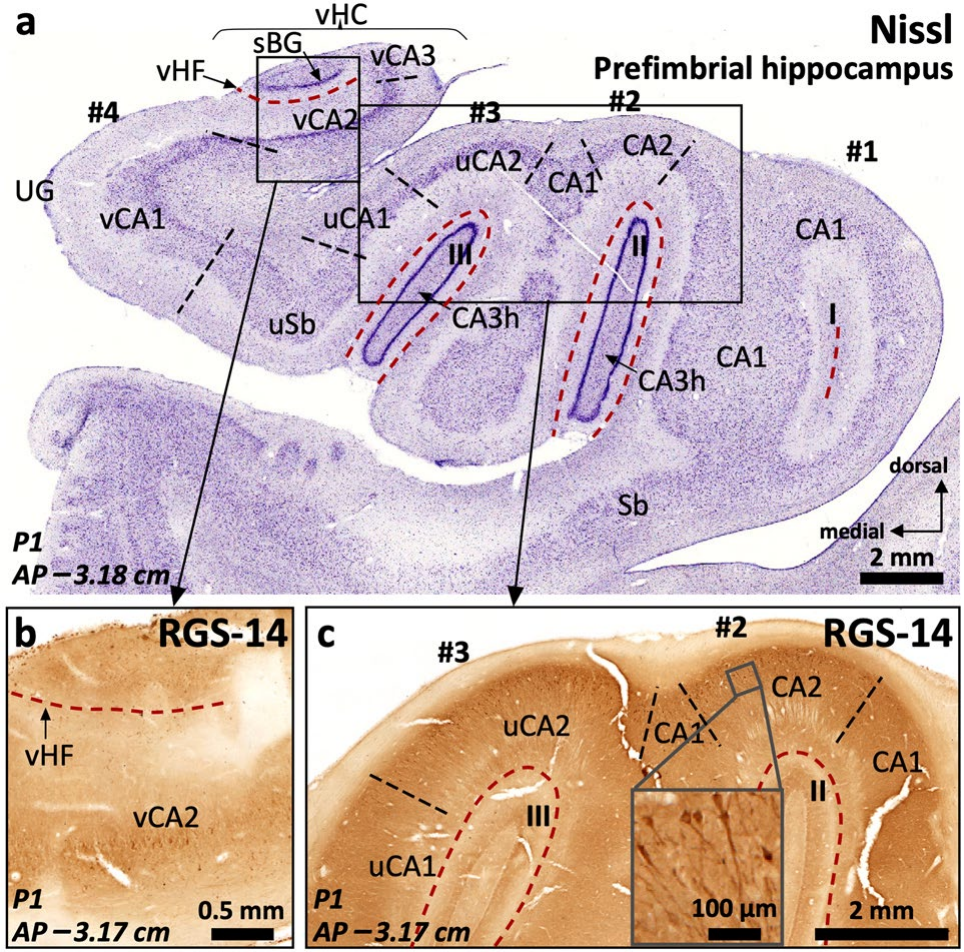
Field delimitation in the human prefimbrial hippocampus combining cytoarchitecture and CA2 immunomarker RGS-14. Molecularly defined CA2 (*i.e.,* RGS-14 + in our context) in the prefimbrial hippocampus is represented in three different medio-lateral levels (**a**). Vertical CA2 (vCA2) corresponding to the vertical hippocampus in the uncinate gyrus (UG) shows dense packing of pyramidal cells (**a**), some of them RGS-14 + (**b**). Laterally (**c**), uncal CA2 (uCA2), corresponding to the third digitation (#3) and its dentate gyrus (III), and a ‘standard’ CA2 (corresponding to the second digitation #2 and its dentate gyrus: II) display dense cytoplasmic immunoreactivity for RGS-14, as well as RGS-14 + apical dendrites (inset in **c**).). Abbreviations: -h as a suffix, hilar; sBG, superior Band of Giacomini; Sb, subiculum; u- as a prefix, uncal; UG, uncinate gyrus; vHC, vertical hippocampus; vHF vertical hippocampal fissure; v- as a prefix, vertical; I, II, III, dentate gyrus of digitations #1, #2, #3, respectively. The code for the broken lines as in Fig. 2b

**Fig. 8:**
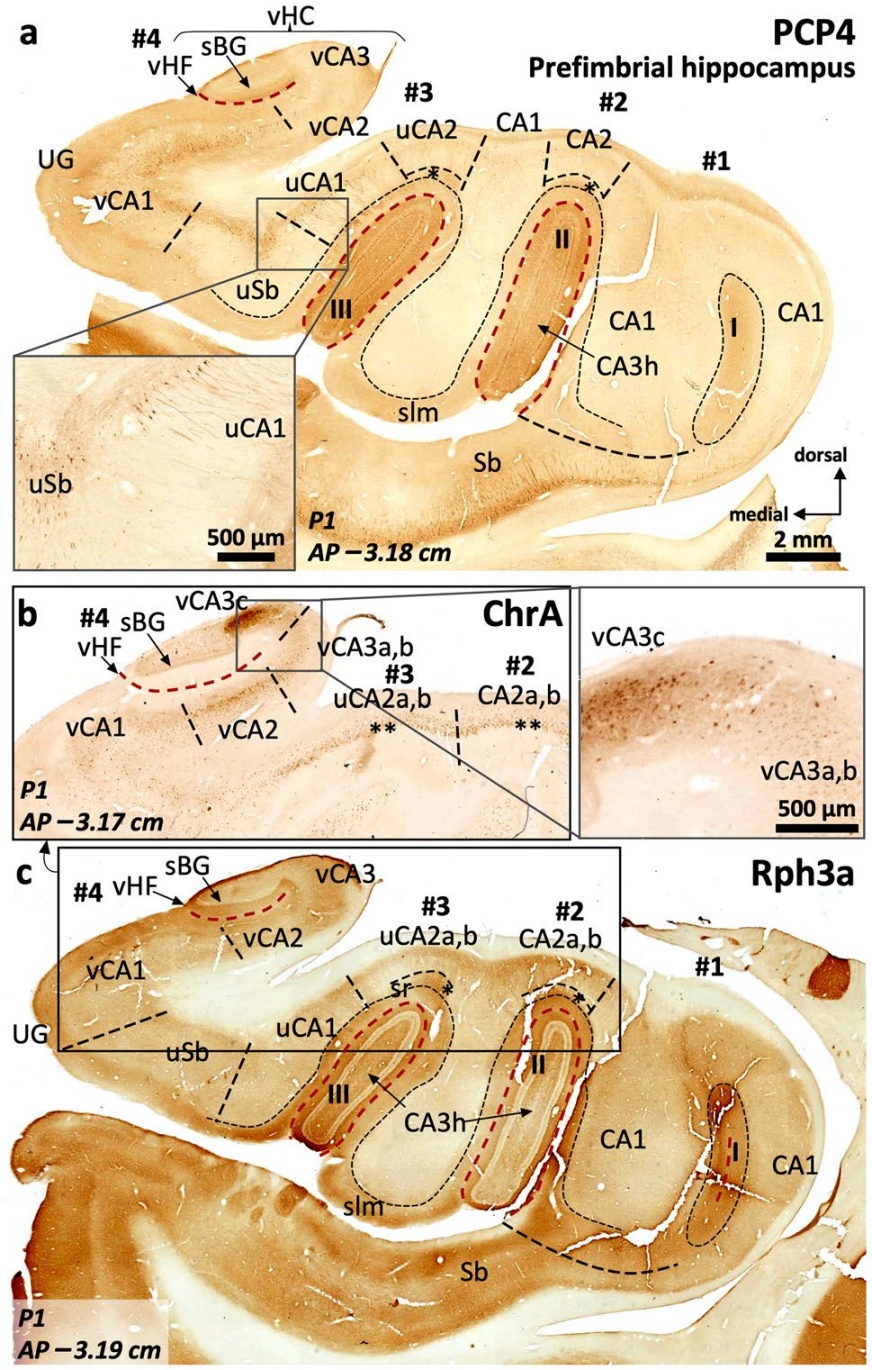
CA2 delimitation and uncinate gyrus – vertical hippocampus (UG, vHC) parcellation in the human prefimbrial hippocampus. The vCA1-vCA2 boundary is characterized by the loss of pyramidal cell PCP4 immunostaining (**a**) and the appearance of a strongly ChrA + pyramidal cell layer in vCA2 (**b**). A lateral vCA3a,b shows weak ChrA immunoreactivity, while the most medial region (CA3c) shows both strong neuropil and cytoplasmic staining in pyramidal cells (inset in **b**). Laterally, a PCP4 + , Rph3a + *stratum radiatum* (* in **a**, **c**) coincides with an elongated CA2 showing ChrA cytoplasmic staining in superficial pyramidal cells (** in **b**). Note that this CA2 is the posterior juncture of uCA2 and CA2 (**b**). In between uCA2 and vCA1, two additional subfields are found: the uncal subiculum (uSb) and a modified subfield with intermediate features between subiculum and CA1; termed uCA1 (**a**, **c**). Inset in (**a**) shows PCP4 + deep pyramidal neurons in uCA1 and uSb, with their apical dendrites stained only in uCA1. Abbreviations: -h as a suffix, hilar; sBG, superior Band of Giacomini; Sb, subiculum; slm, *stratum lacunosum moleculare;* u- as a prefix, uncal; UG, uncinate gyrus; vHC, vertical hippocampus; vHF vertical hippocampal fissure; v- as a prefix, vertical; I, II, III, dentate gyrus of digitations #1, #2, #3, respectively. The code for the broken lines as in Fig. 2b

**Fig. 9:**
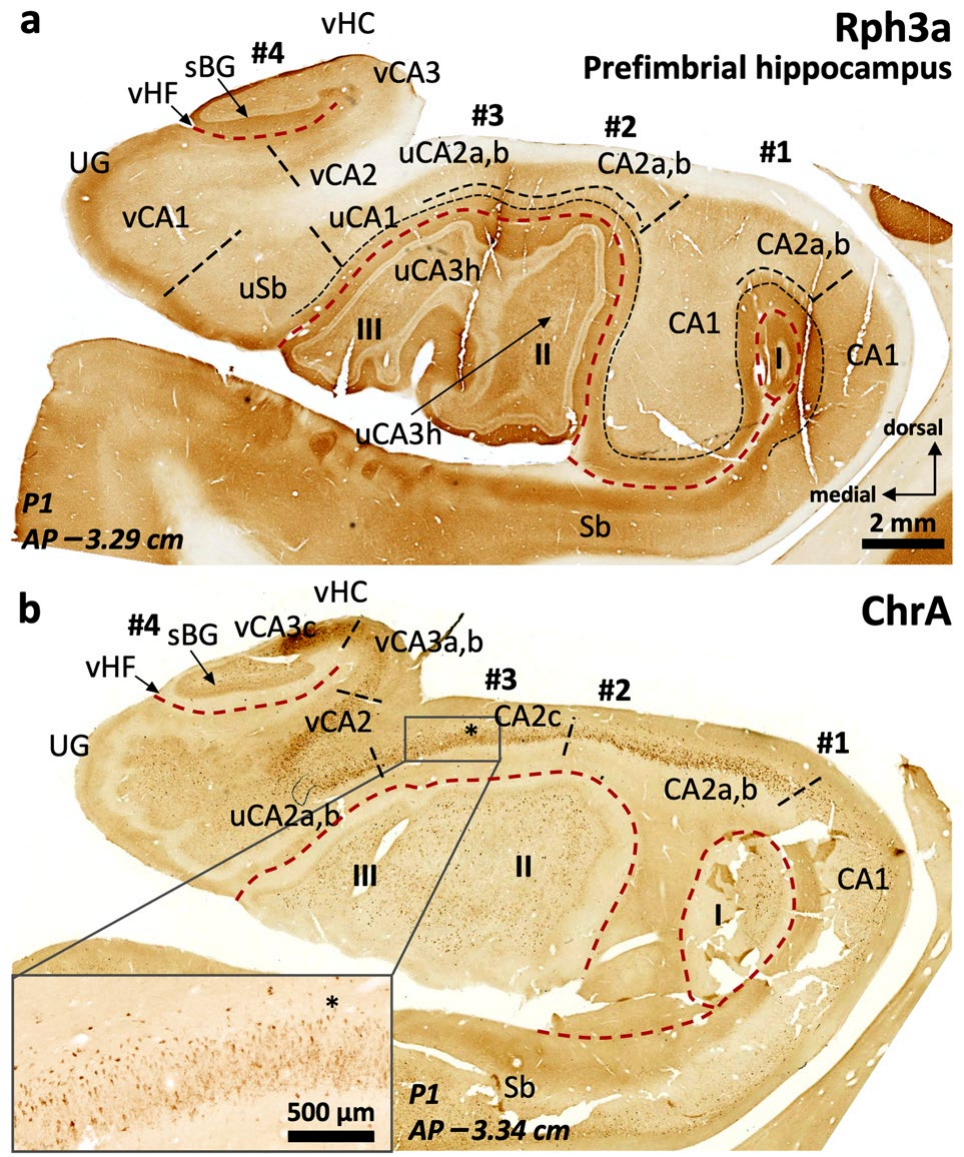
Continuity of anterior CA2 fields. Immediately anterior to the anterior end of the fimbria, CA2 typical and modified fields, including vertical CA2, start to fuse (**a**) forming a continuous band, as visible in (**b**). Note the ChrA + neuronal bodies in pyramidal layer of CA2 (**b**). A zone in CA2c shows lower cytoplasmatic ChrA staining and moderate neuropil staining (inset in **b**: CA2c), which are features similar to the boundary between CA2 and CA3 in the middle hippocampus, and represent the convergence between modified (uCA2, vCA2) and typical CA2. Note the horse hoof-like appearance of the combined hilus of digitations #2 and #3 (II, III). A hair-like artifact in (**b**) has been toned down. Abbreviations: -h as a suffix, hilar; sBG, superior Band of Giacomini; Sb, subiculum*;* u- as a prefix, uncal; UG, uncinate gyrus; vHC, vertical hippocampus; vHF, vertical hippocampal fissure; v- as a prefix, vertical; I, II, III, dentate gyrus of digitations #1, #2, #3, respectively. The code for the broken lines as in Fig. 2b

**Fig. 10:**
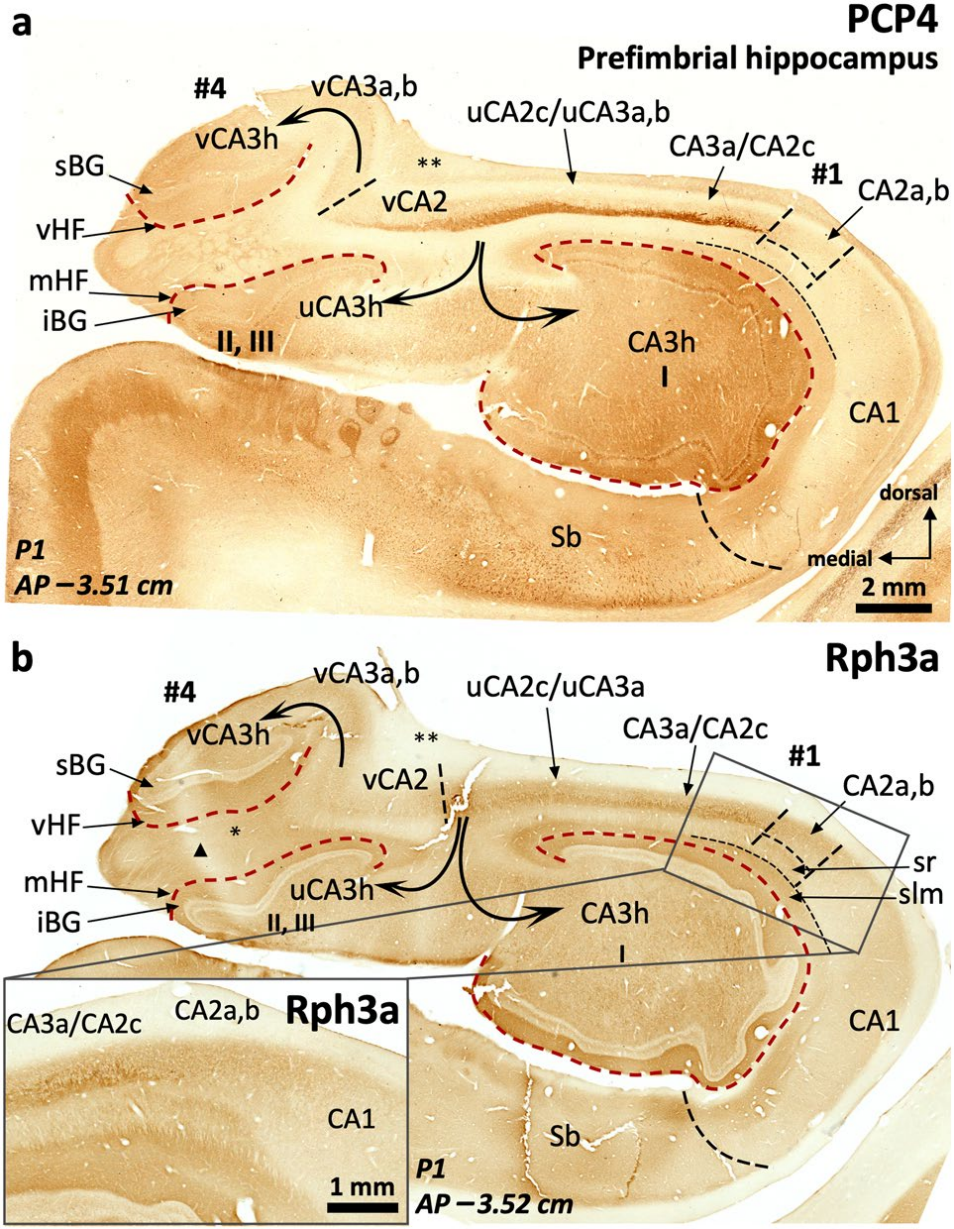
The perifimbrial hippocampus. The hippocampal head is organized around the anterior insertion of the fimbria (**). Beneath the anterior insertion, the pyramidal cell layer bends ventrally to reach the hilar region (curved arrows), where it splits between the hilar region of digitation #1 laterally (I), and the hilar region of the remaining digitations anteriorly and medially (II, III) (digitations #2 and #3 extend well beyond the anterior limit of the fimbria, see Figs. 5, 6, 7, 8, 9). *Stratum lacunosum moleculare* (slm) in the medial end of vCA2 appears expanded (* in **b**) as consequence of tangential sectioning. In this region, CA2 is almost horizontal. Note the vicinity between the vertical hippocampal fissure (vHF) and the medial hippocampal fissure (mHF). Inset in (**b**) shows Rph3a + *stratum radiatum* in CA2, as shown in previous figures. Triangle in (**b**) indicates an artifact: linear-shaped lower Rph3a staining in the *uncus* due to a folding in the tissue while processing. Abbreviations: -h as a suffix, hilar; iBG, inferior Band of Giacomini; mHF, medial hippocampal fissure; sBG, superior Band of Giacomini; Sb, subiculum*;* u- as a prefix, uncal; UG, uncinate gyrus; vHC, vertical hippocampus; vHF vertical hippocampal fissure; v- as a prefix, vertical; I, II, III, dentate gyrus of digitations #1, #2, #3, respectively. The code for the broken lines as in Fig. 2b

**Fig. 11:**
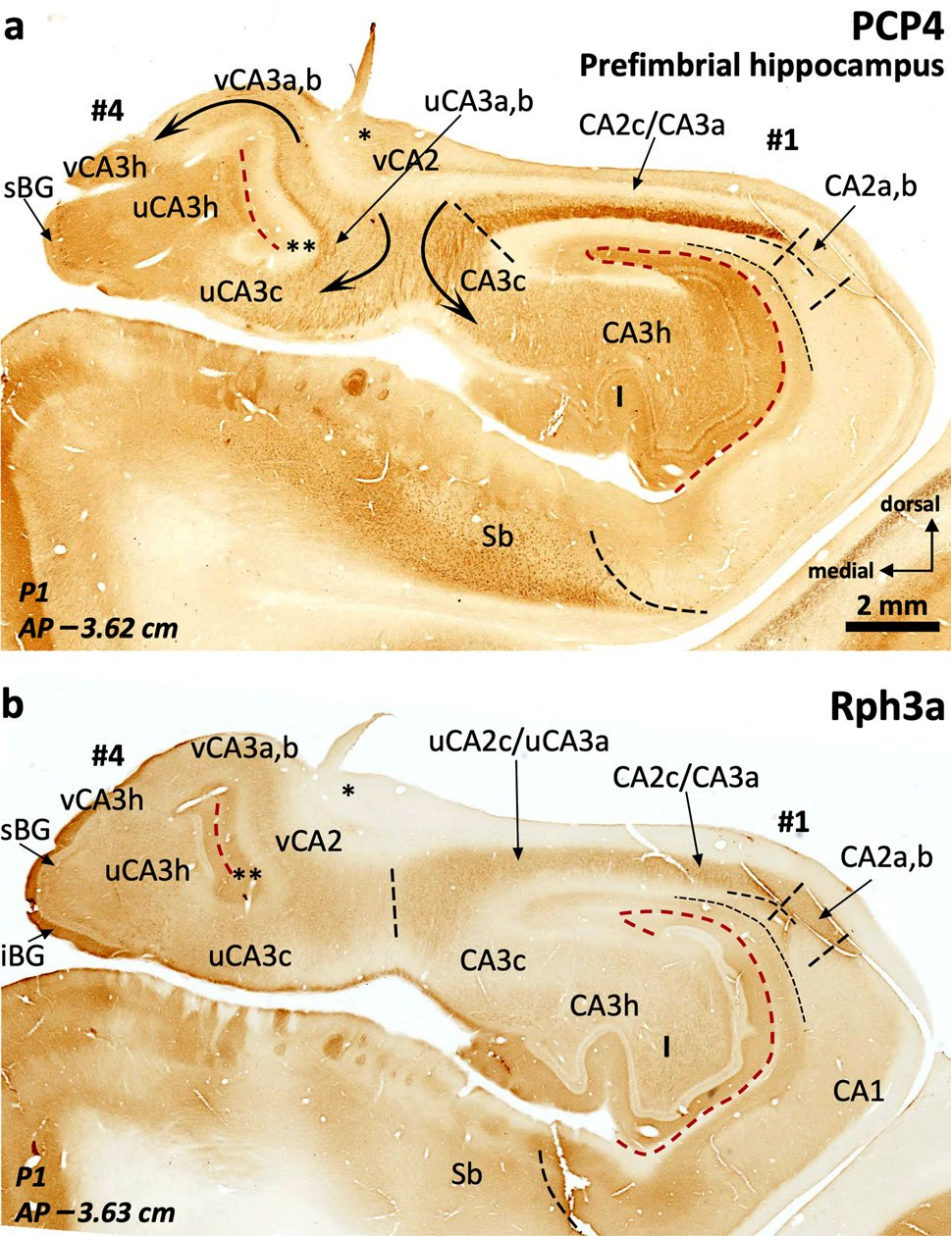
The perifimbrial hippocampus near the uncal apex. The anterior part of the fimbria is indicated by *. The uncal portion of CA3 reaches, from lateral to medial, the hilar region of the vertical hippocampus (left two arrows in **a**). The superior and inferior portions of the Band of Giacomini (i.e., the vertical dentate gyrus; sBG, superior Band of Giacomini; iBG, inferior Band of Giacomini,) are seen as a single, superficial, and non-lobulated dentate gyrus. Note that the medial hippocampal fissure is fused with the vertical hippocampal fissure (**), but there is no communication between this fissure and the non-fissural pial surface. Also, the density of PCP4 + , Rph3a + mossy fibers in the uncal CA3 is quite reduced compared to typical CA3. Abbreviations: -h as a suffix, hilar; Sb, subiculum*;* u- as a prefix, uncal; v- as a prefix, vertical; I, dentate gyrus of digitation #1. The code for the broken lines as in Fig. 2b

The anterior hippocampus is topographically associated to the anterior end of the fimbria. Its medial components are in the posterior part of the *uncus* (Figs. 1a-c, 5). The *uncus* is a hook-shaped topographic division of macrodissected temporal lobe that includes in its posterior region part of the hippocampus (Duvernoy 2005; Swanson 2014). Taking the anterior end of the fimbria as a reference, three distinct subregions are identified in the anterior hippocampus (see Fig. 1a): perifimbrial, prefimbrial and the anterior hippocampal pole, from posterior to anterior (Fig. 5d-g; see also Fig. 1 and Figs. 6, 7, 8, 9, 10, 11). A characteristic feature of the anterior hippocampus is its gyrification consisting in four small gyri named hippocampal digitations. The digitations are labeled from lateral to medial as #1, #2, #3, and #4 (Ding and van Hoesen 2015) (Fig. 5f). The perifimbrial anterior hippocampus (Fig. 5e), which includes the uncal apex, is characterized by a dentate gyrus and a hilus both medial and lateral to the fimbria; the prefimbrial anterior hippocampus (Fig. 5f) is anterior to the anterior insertion of the fimbria (*velum terminale*) and shows a dentate gyrus; finally, the anterior hippocampal pole (Fig. 5g) is the region between the anterior end of the dentate gyri inside the digitations and the smooth transition between the hippocampus and the amygdala. These three regions are described in depth below.

The perifimbrial anterior hippocampus (Fig. 5d, e) includes the lateral (#1) and the medial (#4) digitations separated by the anterior end of the fimbria, which appears as a depression in the superior surface of the anterior hippocampus, lateral to the *uncus*. The lateral digitation (#1) shows a conventional ‘body-like’ hippocampal morphology, with the superficial surface of the dentate gyrus (*margo denticulatus)* as a narrow, denticulate band medial to the parahippocampal gyrus. The medial digitation (#4) includes from superior to inferior the vertical hippocampus (vHC), the uncinate gyrus (UG) and the intralimbic gyrus (IGy; Fig. 5e). The vertical hippocampus is a bulge in the superior surface of the medial digitation and contains a rudimentary dentate gyrus and an obliterated fissure. This dentate gyrus corresponds to the superior Band of Giacomini (sBG; Ding and van Hoesen 2015; Duvernoy 2005; Swanson 2014). The fissure of the vertical hippocampus separates the superior Band of Giacomini from the UG. Inferior to the UG, there is the IGy (Fig. 5e); the UG and the IGy are separated by a hippocampal fissure, which corresponds to the most anterior part of the medial hippocampal fissure (mHF). The IGy also contains a dentate gyrus corresponding to the inferior Band of Giacomini (iBG; Fig. 5e, see also Fig. 10b). Altogether, the Band of Giacomini is a medial component of the uncal *margo denticulatus* (see Fig. 11b and below in this section dealing with the prefimbrial anterior hippocampus). Although some inter-individual variability is present, the IGy forms the uncal *hippocampus inversus* (region where CA3 is superficial, see Duvernoy 2005), which represents the most posteriorly protruding area of the *uncus* or uncal apex (see Supplementary Fig. 2). Remarkably, the fimbria does not directly attach to the uncal apex in our material (Duvernoy 2005), instead it is lateral to it (Fig. 5d, e). As a reference, the fimbrial attachment is located at AP –3.62 (P1) and AP –3.78 (P2), while the uncal apex is situated in AP –3.7 (P1) and AP –3.9 (P2) (Supplementary Table 5).

The prefimbrial anterior hippocampus is located anterior to the perifimbrial part and is the widest anterior hippocampal region. Its main gross morphological features are the absence of a fimbria and the presence of four hippocampal digitations (#1-#4 in Fig. 5f). Digitations #2 and #3 are present only in the prefimbrial hippocampus and the anterior hippocampal pole; they contain a dentate gyrus with their progressively anteriorly-narrowing *margo denticulatus* (MD in Fig. 5f). The lateral digitation (#1) contains a dentate gyrus akin the one present in #2 and #3, albeit shorter in the anteroposterior axis (Fig. 5f, 6, 7, 8, 9). The dentate gyrus is represented twice in the medial digitation (#4): superiorly, in the superior Band of Giacomini, and, inferiorly, in the *uncus* (Fig. 5e, f). The anterior end of the dentate gyrus appears in digitation #2, containing the largest dentate gyrus in the anteroposterior dimension (Figs. 6, 7, 8, 9).

The anterior hippocampal pole (Fig. 5g) is devoid of dentate gyrus and includes the anterior end of the vertical hippocampus and the hippocampo-amygdaloid transitional area (HATA; Fig. 5g). It also includes the most anterior hippocampal fields: the uncal subiculum (uSb) and the uncal CA1 (uCA1) (Fig. 5g), which are further described below.The anterior hippocampal pole is around AP –2.9 (P1) and –3.2 (P2; Supplementary Table 5).

#### Field parcellation of the human anterior hippocampus

Field parcellation in the human prefimbrial hippocampus is shown in Figs. 6, 7, 8, 9. CA1, CA2, and the subiculum are present, including subfields. CA1 and CA2 are present in the most lateral digitations (#1, #2), similar to the CA1 and CA2 fields of the middle hippocampus (Figs. 2, 3, 4, 6, 7). The axis of digitations #2 and #3 contains a dentate gyrus and hilar CA3 (CA3h; Figs. 6, 7, 8, 9), both retaining the immunohistochemical features described for the middle hippocampus (Supplementary Tables 3, 4). In fact, more posteriorly the dentate gyrus of digitations #2 and #3 converge anterior to the fimbria in a single structure (Fig. 9). A slightly PCP4 + and Rph3a + *stratum radiatum*, corresponding to the anterior end of CA2, appears over the dentate gyrus of digitations #2 and #3 (Figs. 6, 7a). This anterior CA2 stands between AP –3.15 (P1) and –3.2 (P2) and is in the same coronal plane as the anterior end of a dentate gyrus molecular layer in digitation #1 (Figs. 6b, 7a, 8c; see the red broken line as a reference). Identification of CA2 in the prefimbrial hippocampus is further validated by the presence of pyramidal neurons immunostained by the *bona fide* markers RGS-14 (Fig. 7b, c) and ChrA (Figs. 8b, 9b). More posteriorly, immediately anterior to the fimbria, CA2 forms a continuous band in the dorsal aspect of the prefimbrial hippocampus (Fig. 9a, b; AP –3.3 in P1, AP –3.6 in P2). In the medial part of the prefrimbial hippocampus, CA2 is present in the medial digitations #3 and #4 (termed uncal CA2, uCA2, and vertical CA2, vCA2, respectively). CA2 of digitation #3 is contiguous medially with a heavily modified subfield that shows intermediate features between subiculum and CA1; this subfield is termed uncal CA1 (uCA1) in agreement with previous authors (Ding and Van Hoesen 2015; Figs. 8a, c, 9a and Supplementary Table 6). Given their singularity, the uncal hippocampal fields merit special attention. The main feature of uCA1 is the presence of a lateral to medial pattern of increasing density of PCP4 + deep pyramidal neurons with their apical dendrites climbing up to the stratum *lacunosum moleculare* (Fig. 8a). The *stratum lacunosum moleculare* of uCA1 also shows dense Rph3a + staining (Fig. 8c). uCA1 borders the uncal subiculum (uSb), located medial to uCA1; uSb also features a deep layer of PCP4 + neurons (Fig. 8a, Supplementary Table 6). The pyramidal layer of the uSb narrows medially, while maintaining its immunohistochemical hallmarks (Figs. 6a, b, 8a, b; see also Fig. 5g). Medial to uSb, there is a vertical CA1 (vCA1), part of the so-called vertical hippocampus. Subfields uSb and vCA1 are present along most of the anteroposterior axis of digitation #4 (Figs. 6, 7, 8, 9).

An interesting feature of the medial digitation (#4) and the UG is the vertical hippocampus, which has unique cytochemical features deserving a specific description (Figs. 6, 7, 8, 9, see also Supplementary Tables 6, 9). The vertical hippocampus contains the following fields: vertical CA1 (vCA1), vertical CA2 (vCA2), vertical CA3 (vCA3) and the superior Band of Giacomini, which is an anteromedial pole of the dentate gyrus. A vestigial vertical hippocampal fissure (vHF, Figs. 5e, f, 6, 7a, b, 8, 9) separates the vertical CA fields and the Band of Giacomini. The dentate gyrus contained in digitation #4 is associated to the convoluted path of the hippocampal fissure (see also Duvernoy 2005, pp. 131–152). vCA1 emerges between the uSb and vCA2; its pyramidal cell layer shows deep PCP4 + neurons (Fig. 8a). vCA2 is formed by densely packed ChrA + , RGS-14 + pyramidal cells (Figs. 7b, 8b). The *stratum lacunosum moleculare* of vCA1 and vCA2 shows Rph3a + neuropil (Fig. 8c). Field vCA3 displays loosely packed pyramidal neurons (Fig. 7a) which are negative to PCP4 and RGS-14 and positive for ChrA (Figs. 8a, 9b); neuropil staining is overall light for these three markers in vCA3a,b while it is strongly positive for ChrA in vCA3c. Near the tip of the UG, this pyramidal cell layer turns ventrally and medially (vCA3c) towards the hilus of the Band of Giacomini, acquiring a unique phenotype with strongly ChrA + (homogenous and non-punctate) pyramidal neurons with no precise orientation and surrounded by a strongly ChrA + neuropil (Figs. 8b, 9b). The vertical hippocampus is approximately at AP –3 in P1 and AP –3.1 in P2. Posteriorly, the vertical hippocampus is continuous with the perifimbrial hippocampus where the Band of Giacomini forms the IGy (Supplementary Tables 5, 9). The histological features of modified hippocampal fields that are specific of the anterior hippocampus are listed in Supplementary Table 6.

**Fig. 12:**
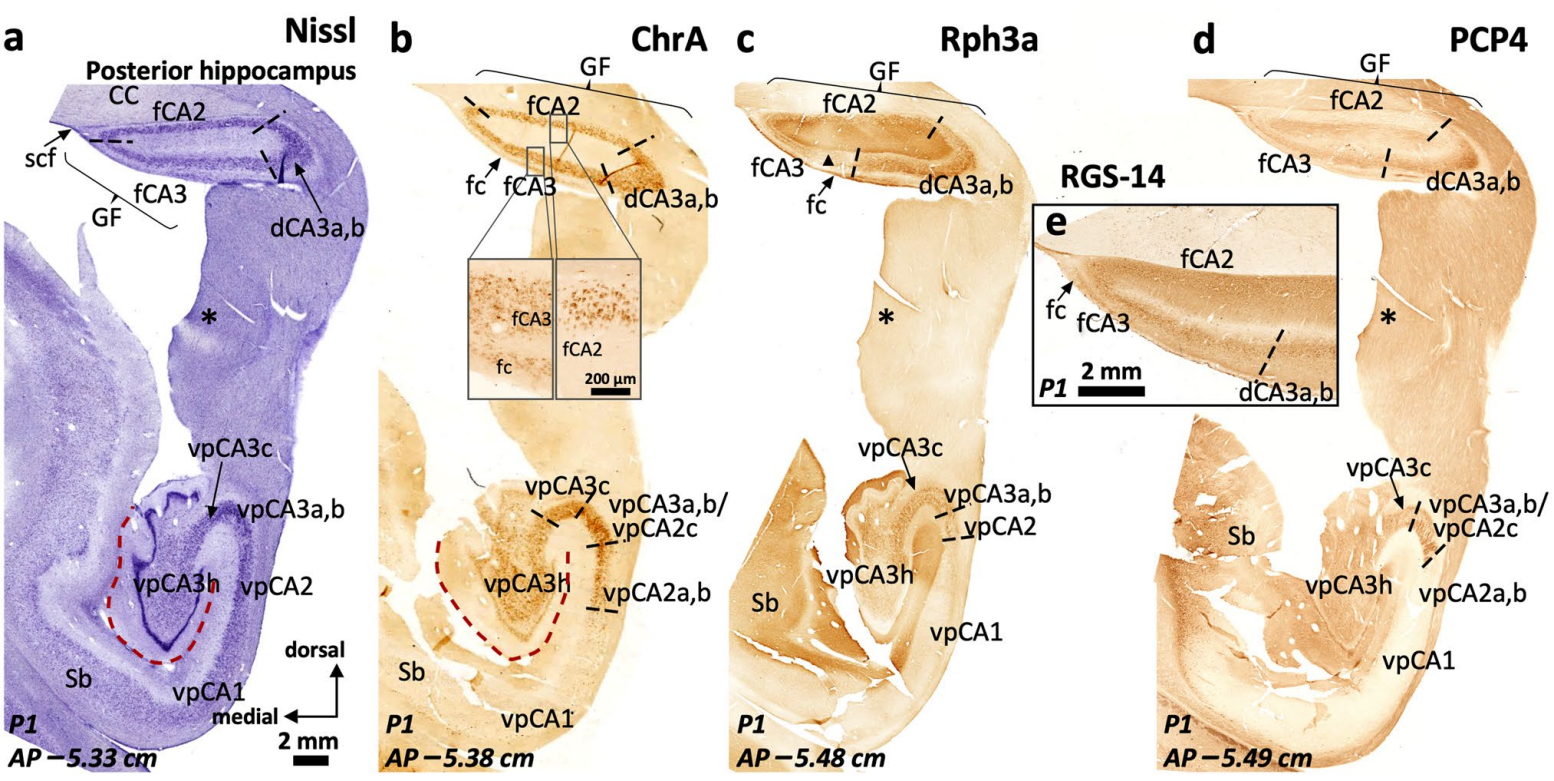
General organization and histochemical features of the posterior hippocampus, the dorsal hippocampus and the gyrus *fasciolaris*. The posterior hippocampus or hippocampal tail is the hippocampal region posterior to the transition between the fimbria and the posterior pillar (*crus*, *) of the fornix. In a coronal section, it is composed by inferior (ventral posterior, vp), superior (dorsal posterior, dp, not present in this figure; see Figs. 13, 14, 15) and posterior proper (posterior, p, not present in this figure; see Fig. 16) fields. The gyrus *fasciolaris* (GF) and the dorsal hippocampus are ventral to the posterior part of the corpus callosum (CC); GF is and separated from CC by the subcallosal fissure (scf). While cytoarchitectonic organization (**a**) of the ventral posterior hippocampus is largely equivalent to the cytoarchitectonic organization of the middle hippocampus (body), there is a lack of correlation between classic cytoarchitectonic landmarks and the distribution of immunohistochemical markers in the gyrus *fasciolaris*, particularly those that define boundaries in the CA2/CA3 region like Chromogranin A (**b**). However, this latter marker allows identification of CA2 and CA3 subfields both in ventral posterior hippocampus and in the gyrus *fasciolaris* (**b**). Note the different patterns of Chromogranin A immunoreactivity in fCA2 and fCA3 (inset in **b**). Neither Rph3a (**c**) nor PCP4 (**d**) are useful to properly demarcate CA2 boundaries, because the CA2 *stratum radiatum* in these regions is not as evident as in anterior regions. Mossy fibers retain their immunohistochemical features (Rph3a + , PCP4 +) in both ventral posterior and dorsal areas, allowing the delimitation of CA3 (vpCA3, dCA3). Dorsal CA3 (dCA3) is continuous with a CA3-like area in the gyrus *fasciolaris* that also shows a deep layer of RGS-14 + pyramidal cells (**e**); this modified fasciolar CA3 field (fCA3) lays underneath a narrow band of dentate gyrus-like tissue corresponding with the *fasciola cinerea* (fc in **b**, **c**, **e;** see Sect. "Field parcellation of the human dorsal hippocampus and the gyrus fasciolaris"). Fasciolar CA2 (fCA2) shows an arrangement of RGS-14 + cells and neuropil that matches the one found in more anterior hippocampal regions (**e**, compare with Fig. 4). Triangle in (**c**) indicates a staining artifact: lower Rph3a immunostaining due to a folding in the tissue while processing. Abbreviations: CC, corpus callosum, d- as a prefix, dorsal; f- as a prefix, fasciolar; GF, gyrus *fasciolaris*; Sb, subiculum; scf, subcallosal fissure; vp- as a prefix, ventral posterior. The code for the broken lines as in Fig. 2b

**Fig. 13:**
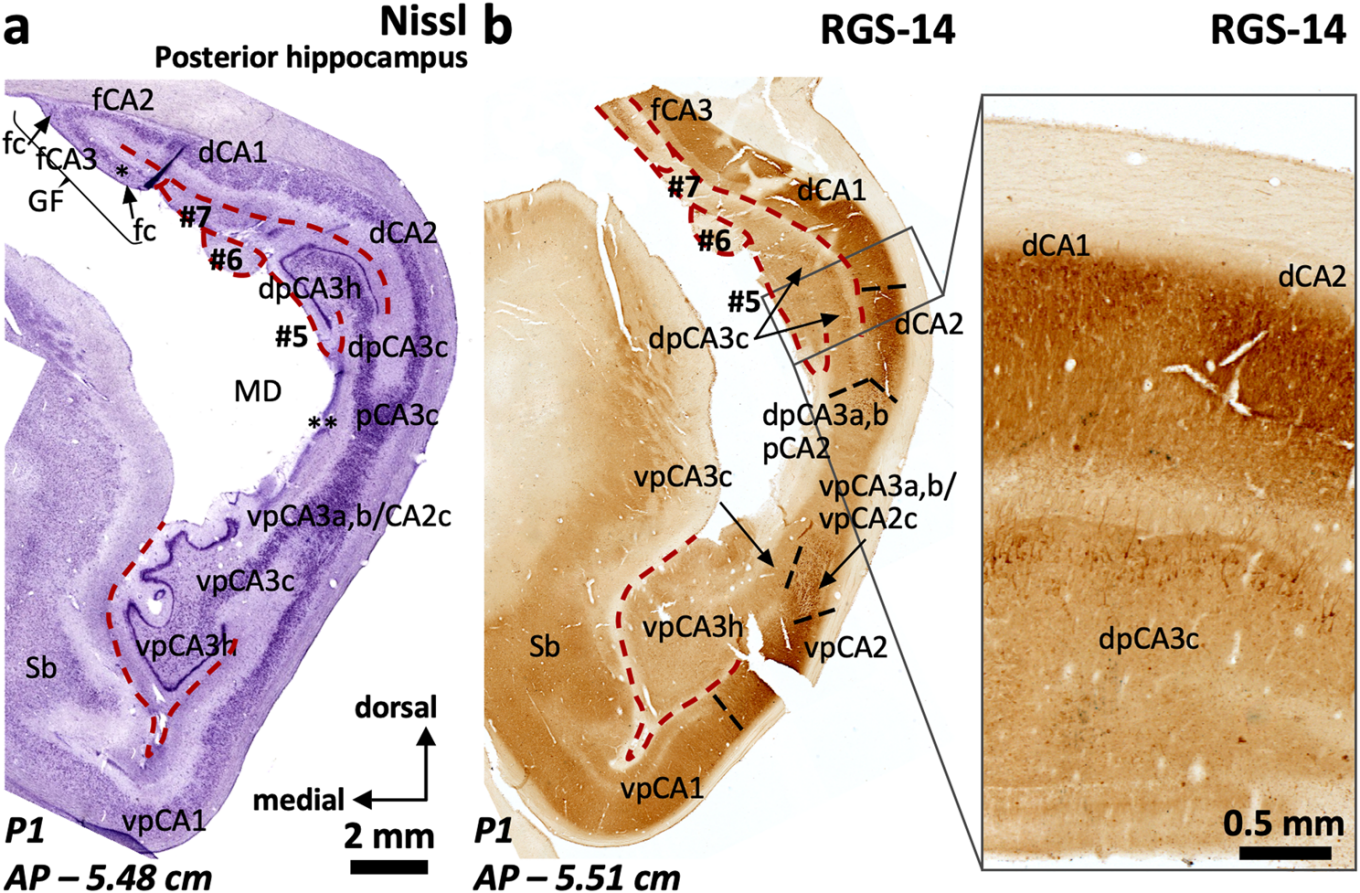
Continuity of posterior hippocampal fields. Immediately lateral and posterior to the posterior attachment of the *crus* of the fornix to the dentate gyrus (**), the posterior hippocampus unfolds its full complexity. An X-shaped structure (**a**) composed by densely packed pyramidal cells organized as two dorsal (dp) and two ventral (vp) blades corresponds to regions with immunohistochemical features resembling typical CA3. This resemblance is more marked in the ventral posterior hippocampus (**b**), where the continuity CA2-CA3a,b-CA3c-CA3h follows a standard staining pattern for RGS-14 (see also Fig. 4). However, dorsal posterior CA3 (dpCA3) shows a deep RGS-14 + pyramidal cell layer (dpCA3c) which is unique of this subfield (inset in **b**). Dorsal CA1 (dCA1) is continuous with a fasciolar CA2/CA3 complex (fCA2, fCA3) that forms a narrow, vestigial, hilus (*) with the *fasciola cinerea* (fc in **a**). Laterally, the most anterior apex of two posterior-to-anterior bulges appear in the medial hippocampal surface, between the *fasciola cinerea* and the dorsal, smooth, component of the *margo denticulatus* (termed #5). In this plane, these bulges are formed mainly by an undefined *stratum moleculare* and are termed #6 and 7# from lateral to medial in keeping with the nomenclature used in the anterior hippocampus. Both correspond to the short gyri of Andreas Retzius (Retzius 1896; Ziogas and Triarhou 2016; ten Donkelaar et al. 2018). Abbreviations: d- as a prefix, dorsal; dp- as a prefix, dorsal posterior; f- as a prefix, fasciolar; GF, gyrus *fasciolaris;* p- as a prefix, posterior; Sb, subiculum; vp- as a prefix, ventral posterior. The code for the broken lines as in Fig. 2b

**Fig. 14:**
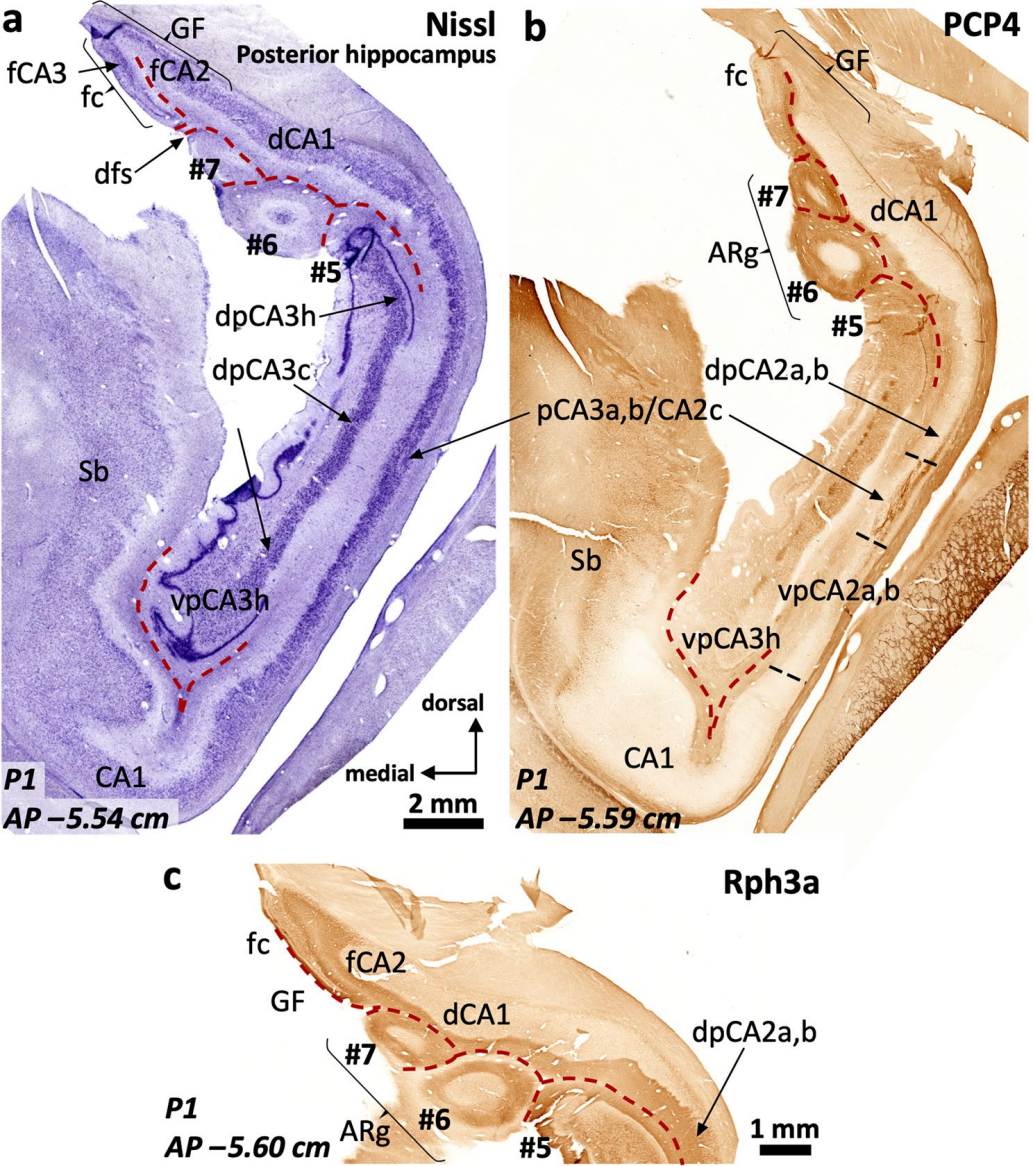
Posterior hippocampus posterior to the *crus*, dorsal hippocampus and gyrus *fasciolaris* (GF). The *fasciola cinerea* (fc) progressively acquires the shape of a minute ‘standard’ dentate gyrus (**a**). It is separated from the medial gyrus of Andreas Retzius (ARg #7) and the gyrus *fasciolaris* (GF) by the dentatofasciolar sulcus (dfs). A remnant from the X-shaped (pCA3a,b) is present in the lateral surface of the posterior hippocampus, displaying PCP4 + mossy fibers (**b**). Rph3a immunostaining (**c**) shows the composition of the Andreas Retzius gyri (ARg): levels anterior to AP –5,5 show no dentate gyrus axis in the ARg; these coronal sections involve only an anterior *cul-de-sac* which is composed of a transversally sliced molecular layer; this molecular layer shows CA1-like features in medial ARg (#7) and subicular-like features in lateral ARg (#6). Abbreviations: d- as a prefix, dorsal; dp- as a prefix, dorsal posterior; f- as a prefix, fasciolar; GF, gyrus *fasciolaris;* p- as a prefix, posterior; Sb, subiculum; vp- as a prefix, ventral posterior. The code for the broken lines as in Fig. 2b

**Fig. 15:**
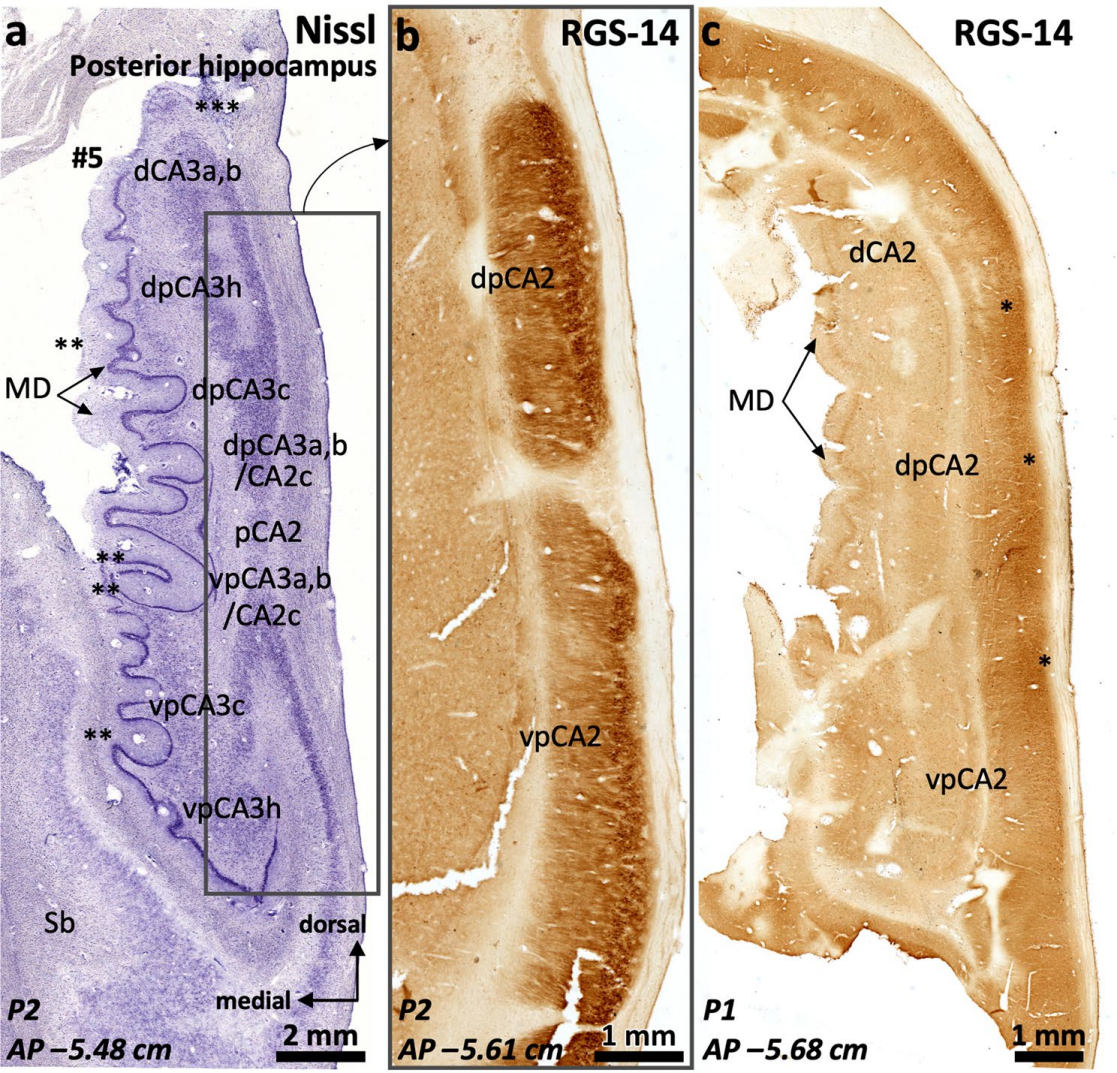
Continuity of posterior CA2 subfields. Posterior to the attachment of the *crus* of the fornix the *margo denticulatus* (MD) spans a long dorso-ventral distance, acquiring in some individuals a remarkably dentate shape (**). Lateral to the X-shaped CA3 complex, a single CA2 field runs parallel to the surface of the MD. This CA2 field is composed by the posterior convergence of vpCA2, dpCA2 and dCA2. Ventral posterior CA2 (vpCA2) shows cytoarchitectonic features (**a**) akin to typical CA2 while dorsal posterior CA2 (dpCA2) and dorsal CA2 (dCA2) show an intermediate architecture between a ‘canonical’ CA1 and a typical CA2. However, all three CA2 fields show a deep layer of RGS-14 + pyramidal cells (**b**, * in **c**), therefore matching the definition of molecularly defined CA2. A presumptive *fasciola cinerea* appears attached to the dorsal surface of the dorsal posterior hippocampus (*** in **a**). Abbreviations: d- as a prefix, dorsal; dp- as a prefix, dorsal posterior; p- as a prefix, posterior; Sb, subiculum; vp- as a prefix, ventral posterior

Immediately anterior to the fimbrial insertion (i.e., around AP –3.50), almost the whole dorsal surface of the hippocampal head is composed by a rim of CA2 (Fig. 9; from medial to lateral vCA2-uCA2-CA2). Posterior to this level, the perifimbrial anterior hippocampus digitations #1 and #4 are separated by the fimbrial attachment. They share an hilus linked by a continuous CA3 underneath the fimbria, encompassing vCA3-uCA3-CA3 and showing an X-shape (AP –3.5 in P1; AP –3.75 P2), a landmark that also exists in the posterior hippocampus (Figs. 10, 11; see also Figs. 12, 13, 14, 15 and particularly 13a, 15a and Supplementary Figs. 2, 3d). The lateral digitation (#1) is continuous with the middle hippocampus and shows a similar field parcellation. Digitation #4 contains an uncal CA3 (uCA3) with typical immunohistochemical features, though with less remarkable PCP4 + , Rph3a + mossy fibers. In this medial digitation (#4), the dentate gyrus corresponds with the Band of Giacomini. The Band of Giacomini is an anterior representative of a dentate gyrus and, as such, forms its own gyrus, which corresponds inferiorly to the IGy (Figs. 10, 11, Supplementary Fig. 2, Supplementary Table 9). Both digitations (#1 and #4) share a common hilus at the prefimbrial hippocampus. See Supplementary Fig. 3 for a 3-D reconstruction of this region.

The Band of Giacomini is present as a single structure posterior to the fimbrial attachment (AP –3.6 in P1 and AP –3.8 in P2) and is separated from uCA1 in digitation #4 by the hippocampal fissure. The particular morphology of the Band of Giacomini and the dentate gyrus of digitation #4 runs in parallel to the hippocampal fissure (Figs. 10, 11, Supplementary Fig. 2), which circa AP –3.6 (P1) to AP –3.8 (P2), curves laterally and is present deep and convoluted into the uncal parenchyma (red broken lines Figs. 10, 11). The *stratum moleculare* around the convergence of vCA2 and uCA2 is tangentially cut in coronal sections and interrupts the Band of Giacomini that forms two bulges in digitation #4, the sBG and iBG (asterisk in Fig. 10b). The hilar region of the Band of Giacomini corresponds to the convergence of vCA3 and uCA3, as supported by the expression of PCP4 and Rph3a (see Fig. 11). The posterior end of the Band of Giacomini granule cell layer is around AP –3.6 (P1) and AP –3.8 (P2). The uncal apex is formed by the molecular layer of the Band of Giacomini in its medial edge and by an outgrowth of uCA3 in its ventrolateral surface (Supplementary Fig. 2). The morphology of uCA3 is variable between cases; in some of them (I1, P2) it is narrow enough that a deep notch is present between digitations #4 and #1 (*e.g.,* case I1 in Fig. 5e).

### The human posterior hippocampus, dorsal hippocampus and the gyrus fasciolaris

#### Macroscopic anatomy and nomenclature

The posterior hippocampus lies posterior to the transition of the fimbria and the posterior pillar (*crus*) of the fornix (Fig. 1a). The subregions of the posterior hippocampus have received many names (Duvernoy (2005); we use here a simple, internally consistent, terminology. It is of note that we do not identify an intralimbic gyrus in the posterior hippocampus; we restrict the concept of intralimbic gyrus to the *uncus* (in the anterior hippocampus) to avoid terminological confusion (Insausti et al. 2010; Ding and van Hoesen 2015). The posterior hippocampus can be distinguished from the middle hippocampus in the temporal lobe at AP –5.2 (in both cases P1 and P2), and is recognized under the posterior part of the corpus callosum between AP –5.2 (P1) and AP –5.4 (P2) (see further details in Supplementary Table 7). Dorsal to the posterior hippocampus are the dorsal hippocampus and the gyrus *fasciolaris*, underneath the ventral part of the posterior corpus callosum (Fig. 1e).

#### Field parcellation of the human posterior hippocampus

**Fig. 16:**
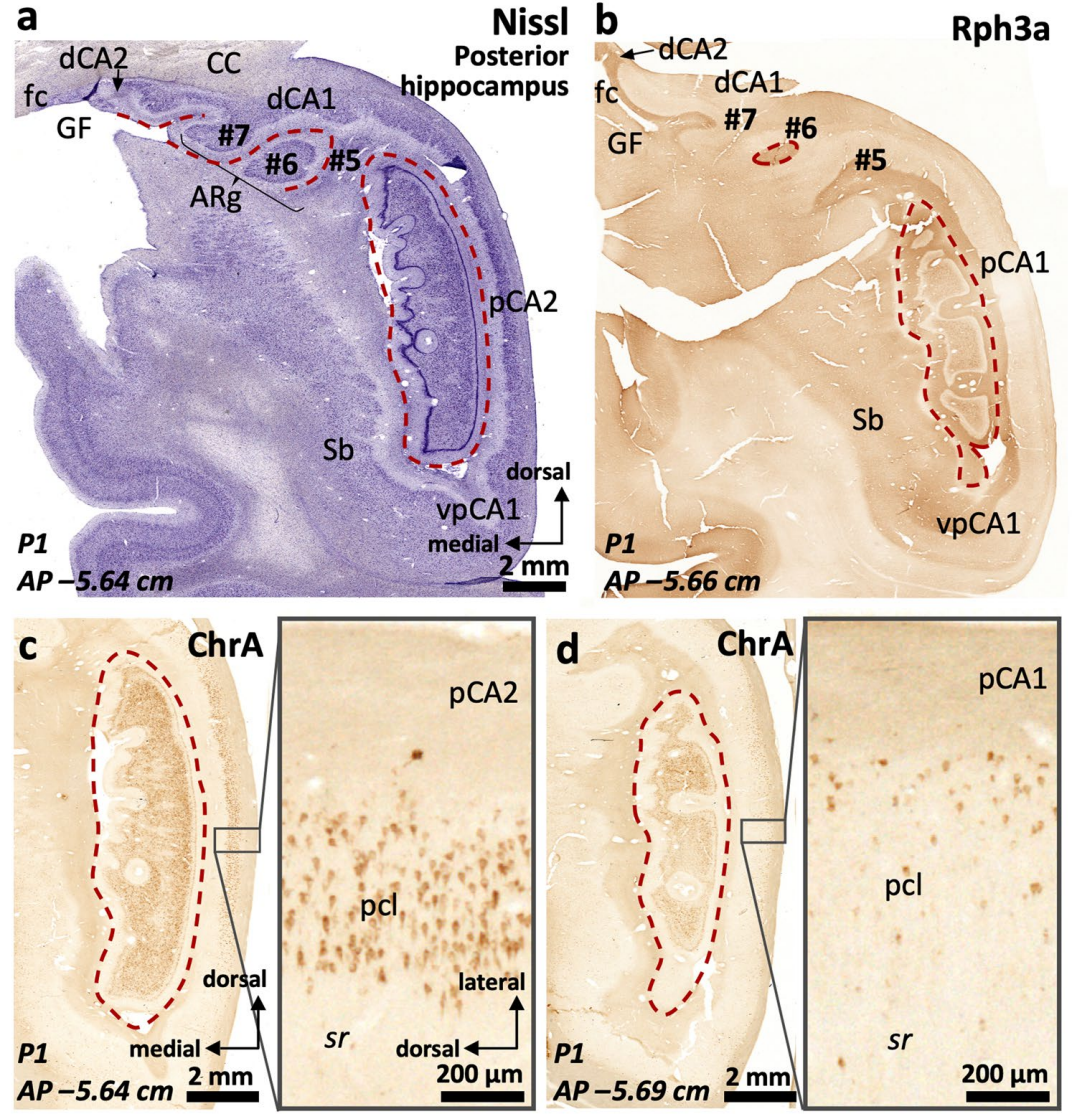
Cytoarchitectonic organization around the posterior end of the dentate gyrus. A single pCA2 evolves into a subcallosal dorsal CA1 (dCA1) that is connected to the *fasciola cinerea* (fc) through a very narrow dCA2/CA3 complex. From dCA1 (**a**) two short-based convolutions emerge, bulging in the ventromedial surface of the dorsal hippocampus (Andreas Retzius gyri, ARg, #6 and #7). The most lateral (#6 in **b**) shows an internal core composed of dentate gyrus-like molecular layer. This is highlighted with Rph3a in **b**, with the red dashed line representing the limit of the molecular layer surface of the dentate gryus. Note in **c** and **d** the abrupt transition between a posterior CA2 and a posterior CA1, highlighted by the loss of ChrA + pyramidal cell bodies in the pyramidal cell layer (pcl). Abbreviations: CC, corpus callosum*;* d- as a prefix, dorsal; GF, gyrus *fasciolaris*; p- as a prefix, posterior; Sb, subiculum; sr, *stratum radiatum*; vp- as a prefix, ventral posterior. The code for the broken line as in Fig. 2b

The posterior hippocampus is composed by three distinct regions (Fig. 1e): an inferior one (ventral posterior hippocampus, vp in Figs. 12, 13, 14, 15), a superior one (dorsal posterior hippocampus, dp in Figs. 13–15), and a posterior proper (posterior, p in Fig. 16) which is only present at the most posterior levels and is formed by posterior CA2 (pCA2), posterior CA1 (pCA1) and subicular fields (Figs. 13, 14, 15, 16).

The ventral posterior hippocampus (vp in Figs. 12, 13, 14, 15) shares immunohistochemical features with the middle hippocampus. Ventral posterior CA3 is formed by progressively larger and more packed pyramidal cells arranged in three distinct subfields (Fig. 12a): a) hilar CA3 (vpCA3h); b) CA3c (vpCA3c), showing diffuse fiber immunoreactivity to PCP4 and Rph3a (Fig. 12c, d); c) vpCA3a,b/vpCA2c with a Rph3a + , PCP4 + *stratum lucidum* (Figs. 12c, d, 14b), and a ChrA + , RGS-14 + pyramidal cell layer (Figs. 12b, 13a, b). Ventral posterior CA2a,b (vpCA2a,b) has a ChrA + , RGS-14 + pyramidal cell layer (Figs. 12b, 15b), like CA2 in the middle hippocampus.

In the posterior hippocampus, CA2 links vpCA3 and dpCA3 forming an X-shaped structure (Fig. 13a). Posterior CA2 (pCA2) shows strongly RGS-14 + , ChrA + deep pyramidal neurons whereas the superficial ones are RGS-14–, ChrA + (pCA2 in Fig. 16c). The anterior boundary of pCA2 can be set by the presence of Rph3a + , PCP4 + mossy fibers (Fig. 14b). Posterior CA2 (pCA2, Figs. 15, 16a, c) is continuous with posterior CA1 posteriorly (pCA1 in Fig. 16b, d). Posterior CA1 shows scattered ChrA + neurons, a feature similar to CA1 in the middle hippocampus (Fig. 16d; see also Fig. 3a, d). Dense RGS-14 + neuropil is also a feature of pCA1 and pCA2 pyramidal cell layers; RGS-14 + neuropil staining gradually wanes posteriorly, with no clear boundary with the subiculum (Fig. 13b). The posterior limit of pCA1 is at AP –5.7 (P1) and AP –5.85 (P2); it is posteriorly continuous with the posterior subiculum, which represents the posterior end of the hippocampal formation (AP –5.9 in P1 and AP –6.0 in P2, see Supplementary Table 7).

The dorsal posterior hippocampus, which is shorter in the anteroposterior axis than the ventral posterior hippocampus, shows some different cytoarchitectonic and cytochemical features as compared to the ventral posterior hippocampus. The density of the pyramidal cell layer is higher in dpCA2c/CA3a,b and dpCA3c than in their ventral counterparts; the deepest layer of dpCA3c shows RGS-14 + pyramidal cells (Fig. 13a, b; Supplementary Table 8).

Throughout the whole posterior hippocampus, the surface of the dentate gyrus (the *margo denticulatus*) is highly variable (Figs. 13, 14, 15, 16). Contrary to the Band of Giacomini (of the anterior hippocampus) and the *fasciola cinerea*, which are smooth bands of dentate gyrus in all brains, the morphology of the dentate gyrus in the posterior hippocampus differs among individuals: in some individuals prominent curls predominate (*e.g.,* P2; Fig. 15a), while in others, smoother ones are present (*e.g.,* P1; Fig. 14a). The dentate gyrus curls are mostly evident in, but not restricted to, the posterior hippocampus. Interestingly, the curls contain a granule cell layer and a fully developed molecular layer with Rph3a + , PCP4 + , ChrA– neuropil surrounding a perforating artery (Figs. 14a, b, 15a, c, 16). The dentate gyrus curls have been named “dentations” by Fleming Beattie et al. (2017).

#### Field parcellation of the human dorsal hippocampus and the gyrus fasciolaris

The dorsal hippocampus lies dorsal to the dorsal posterior and the posterior hippocampus. The cytoarchitectonic and cytochemical features of the dorsal hippocampus are similar to those of the dorsal posterior hippocampus. Dorsomedial to the dorsal hippocampus is the gyrus *fasciolaris* (Figs. 1e, 12, 13, 14). Both the dorsal hippocampus and the gyrus *fasciolaris* are ventral to the posterior end of the corpus callosum.

The gyrus *fasciolaris* is a very thin extension of the hippocampal region around the splenium of corpus callosum (Swanson 2014). It contains the *fasciola cinerea* and two ammonic fields (fCA2 and fCA3) that surround the *fasciola cinerea* (Figs. 12, 13, 14, Supplementary Fig. 4). The *fasciola cinerea* is a vestigial dentate gyrus. The dentatofasciolar sulcus separates the gyrus *fasciolaris* from the ammonic fields of the gyrys *fasciolaris* and from the most dorsal Andreas Retzius gyrus (see below) (Fig. 14a, Supplementary Fig. 4). The dentatofasciolar sulcus adjoints the hippocampal fissure at AP –5.38 (P1) and approximately AP –5.45 (P2) (Fig. 14a, Supplementary Fig. 4c). The *fasciola cinerea* has a medial blade, which is a rudimentary structure (**** in Supplementary Fig. 4c).

The anterior end of the gyrus *fasciolaris* is composed by fCA2 and fCA3 (Fig. 12, Supplementary Fig. 4), forming a posterior *hippocampus inversus* like the one described in the *uncus*. This anterior end is at AP –5.23 (P1). It should be noted that the entire gyrus *fasciolaris* was hardly evident in case P2; it was located at circa AP –5.4 to –5.5.

Fasciolar fCA2 is populated by densely packed ChrA + cells within a RGS-14 + neuropil (Fig. 12b, e). Fasciolar fCA3 pyramidal cells show some punctate cytoplasmic immunoreactivity to ChrA, while ChrA + boutons are widespread around the pyramids (Fig. 12b). Fasciolar fCA3 pyramidal layer is stratified with a deep RGS-14 + pyramidal cells, similar to that in dorsal CA3 (Fig. 12e). The boundary between fCA3 and dorsal CA3a,b is marked by the absence of Rph3a + , PCP4 + mossy fibers in fCA3 (Fig. 12c, d).

Lateral (case P1) or inferolateral (case P2) to the dentatofasciolar sulcus, there are bumps in the ventral surface of the dorsal hippocampus (Figs. 13, 14, 15, 16, Supplementary Fig. 4). These bumps have been called Andreas Retzius gyri (Retzius 1896; Ziogas and Triarhou 2016; ten Donkelaar et al. 2018; see also Chang et al. 2018). Their numbers and structure are variable, ranging from vestigial structures to fully developed (almost pediculated) extrusions. Fully developed Andreas Retzius gyri are composed by a core of dentate gyrus surrounded by CA1 (Supplementary Fig. 4d, e). Their anterior and inferior aspects, therefore, correspond to the *stratum lacunosum moleculare* of CA1 (Fig. 16a).

## Discussion

We propose here a parcellation of the human hippocampus along its full longitudinal axis based on distinct immunohistochemical features and providing stereotaxic coordinates in the coronal plane. Findings of this study are of interest for pathologists analyzing hippocampal biopsies or necropsy specimens, as well as for neuroradiologists. We first define the features of main hippocampal fields in the middle hippocampus, and then analyze them in the anterior and posterior hippocampus, which have a more complex structure. In the anterior hippocampus, we provide descriptions of some modified subfields located in the *uncus* that have no precedent in the literature (Supplementary Tables 5, 6) and are not identifiable using protocols solely based on cytoarchitectural traits (Williams et al. 2023). To the best of our knowledge, this is the first study providing a parcellation of the posterior hippocampus (Supplementary Tables 7, 8). This work deals mainly with the field parcellation of the hippocampus proper, and does not deal with intrinsic parcellation in other regions of the hippocampal formation like the subiculum (Ishihara et al. 2020; Williams et al. 2023).

### Methodological issues

Parcellation of the human hippocampus *in-vivo* is an evolving subject that is frequently limited by the lack of histologically based countercheck. The use of a stringent method for tissue collection and sectioning allowed us to provide AP coordinates for our data (Talairach and Tournoux 1988; García-Cabezas et al. 2023), as well as histological boundaries that are related to brain macroscopic landmarks (*e.g.,* the fimbrial attachment). The combination of immunohistochemical markers identified here provides better regional, field, and subfield discrimination in critical regions like the anterior or posterior hippocampus as compared to protocols based on cytoarchitecture, and earlier immunohistochemical markers (Ding and Van Hoesen 2015).

This work is intended to be a tool for researchers and clinicians; therefore, we have set criteria valid for tissue of different origins, wide ranges of *post-mortem* delays, fixation methods, and processing protocols (McFadden et al. 2019). Detailed description of the hippocampus has been performed in two gold standard brains fixed in paraformaldehyde by transcarotid/transvertebrobasilar *ex-situ* perfusion (García-Cabezas et al. 2007, see also Terreros-Roncal et al. 2022). This method allowed for steady, homogeneous, fixation and provided immunogenicity virtually equivalent to *ex-vivo* samples (Adickes et al. 1997; Lyck et al. 2008; Lavenex et al. 2009). However, no fixation protocol is free from shrinkage (McFadden et al. 2019), neither is our cryoprotection protocol (*e.g.,* Estrada et al. 2017; McFadden et al. 2019). Nevertheless, providing stereotaxic information relative to the anterior commissure to histological specimens is a unique and practical approach for brain imaging correlations. Remarkably, the AP stereotaxic values for brains P1 and P2 were consistent.

The battery of markers selected here as suitable for field parcellation (PCP4, Rph3a, RGS-14, ChrA) allowed reliable border tracing for all CA fields including subfields such as CA2a-c. Unfortunately, these makers do not allow to trace limits of subfield borders within the subicular complex (*e.g.,* prosubiculum-subiculum), where histological segmentation relies on a combination of cytoarchitectonic features (Williams et al. 2023). The border between CA1 and subiculum was marked by deep PCP4 + pyramidal cells in the subiculum; the histological resolution of this border may be strengthened by the analysis of chromophilia, neuron size and collinearity (as recently described by Williams et al. 2023; see also Chevaleyre and Siegelbaum 2010; Ito and Schuman 2012; Ding and van Hoesen 2015; Benoy et al. 2018). Secretagonin (Tapia-González et al. 2020) and neurotensin (Bienkowski et al. 2021) are additional useful markers of the human CA1-subiculum border.

### Histological studies using stereotaxic sampling methods are needed for adequate hippocampal segmentation

Lesions in the hippocampal formation are known to be involved in the pathogenesis of many diseases like epilepsy (Dam 1980; Mathern et al. 1996; Thom et al. 2005), schizophrenia (Benes et al. 1998), depression and bipolar disorder (Knable et al. 2004), and Alzheimers’s disease (Dickson et al. 1991), among others. Given the limited availability and inherent difficulties of human tissue sampling, wide scale *in-vivo* analysis through neuroimaging is typically used to detect early hippocampal neurodegenerative processes.

Recent developments in MRI technology allow for the detailed study of the hippocampus (Yushkevich et al. 2009; Kerchner et al. 2010). However, most protocols for hippocampal segmentation (either automatic, semi-automatic or manual) are unable to fully distinguish precise field borders (see Yushkevich et al. 2015 for a review on the matter), usually amalgamating fields in groups such as CA2/CA3 (*e.g.,* van Leemput et al. 2009) or CA3/CA4/DG (Steve et al. 2020). Few protocols provide clear distinction between CA3-CA2-CA1 (Wang et al. 2003; Mueller et al. 2007; Kerchner et al. 2012; Yushkevich et al.  2015; Pressner and Schoemaker, unpublished work cited by Yushkevich et al. 2015; Wisse et al. 2017). Indeed, the medial limit of CA3 is difficult to determine (Mueller et al. 2007; Kerchner et al. 2012), except for 7 T MRI (Parekh et al. 2015). Moreover, field distribution in the anterior hippocampus lacks adequate definition (*e.g.,* Zeidman et al. 2015; particularly useful is the comparative analysis of segmentation protocols archived as supplementary material in Yushkevich et al. 2015). Furthermore, the posterior hippocampus is frequently unmentioned or combined as a single structure using the posterior pillars of the fornix as a reference (*e.g.,* Van Leemput et al. 2009).

Precise morphological distinction between fields and subfields in the human brain, particularly in the hippocampus, is necessary for the implementation of reliable computational models, which presently rely on diverse datasets of human histological samples stained with nonspecific methods, as well as on morphological data from rodents (see DeKraker et al. 2020; Gandolfi et al. 2023). Even when protocols are partially based on intra-study histological cross-checks or *post-mortem* tissue analyses (Adler et al. 2014, 2018; Iglesias et al. 2015; Steve et al. 2017), subfield delimitation has not been performed and field delimitation is based upon gross morphological landmarks. The present study provides morphologists with novel data for detailed field parcellation. The present data, that include stereotaxic coordinates (see Supplementary Tables 5, 7), may serve also as templates for neuroimaging segmentation. Furthermore, the present study includes previously understudied regions (Supplementary Table 9) and features, like hippocampal dentation in the hippocampal tail (see Fleming Beattie et al. 2017). As our sample is mostly based on two reference cases, interindividual variability should be evaluated by adding more cases; all the more so regarding the posterior hippocampus and gyrus *fasciolaris*, where interindividual variability is most notable. The *indusium griseum* has not been included in our study (see Sanders et al. 2021).

Subfield delimitation is additionally relevant. It has been shown that the ‘receptorarchitecture’ of each hippocampal field varies along its axis (Palomero-Gallagher et al. 2020). On this point, studies analyzing neurotransmitter receptor densities and gene expression levels in the same samples (Zhao et al. 2023) may benefit from the present data because they allow for subfield-wise tissue collection. Remarkably, our data confirm medio-lateral heterogeneities within each subfield, match the subfield receptor densities data in CA3 (Palomero-Gallagher et al. 2020), and provide a neurochemical correlate for the known divergence of modified subfields (e.g., uCA2) in the anterior hippocampus (Ding and Van Hoesen 2015; Williams et al. 2023).

### Rationale for identifying hippocampal regions and fields

We propose fimbrial features as landmarks for identifying hippocampal regions in the human brain: the anterior hippocampus is located at the *velum terminale* (fimbrial anterior insertion), and anteriorly. The fimbria has a horizontal orientation along the middle hippocampus. The fimbria becomes vertical (*crus* of the fornix) at the posterior hippocampus. The *velum terminale* of the fimbria is also a proxy for the identification of the IGy (see Fig. 5e), which is a landmark for the transition between the middle and anterior hippocampus in neuroimage (Olsen et al. 2019). The hilus of the IGy is continuous with the medial branch of CA3 that forms a characteristic x-region (see de Flores et al. 2020 and, particularly, Williams et al. 2023). The use of the fimbria, which is identifiable both in macrodissection and neuroimage, as a guidepost for regionalization and parcellation eases the macro-microscopic correlations and simplifies region and field identification.

Using morphological criteria different from the present ones, Williams et al. (2023) have proposed recently a parcellation of the anterior hippocampus in eight regions: *genu*, *genu-pes*, *pes*, *pes*-dentate gyrus, full dentate gyrus, separated dentate gyrus, x-region, and *uncus*-body. The *genu*, *genu*-*pes* and *pes* of Williams et al. (2023) correspond to the present anterior hippocampal pole. The *pes*-dentate gyrus of Williams et al. (2023) corresponds to the present prefimbrial hippocampus; and the full-dentate gyrus, separated dentate gyrus, x-region and *uncus*-body are within the present perifimbrial hippocampus. The present description of the anterior hippocampus largely overlaps with those of Duvernoy (2005) and Ding and Van Hoesen (2015) (see also Insausti and Amaral 2004, for the macroscopic aspects). A divergence is the present identification of the dentate gyrus of the vertical hippocampus with the superior Band of Giacomini, based on immunohistochemical patterns (in contrast to the radiological imaging and macroscopic observations predominant in Duvernoy’s proposal). It is of note that the continuity of the vertical hippocampus in the *uncus* and the Band of Giacomini is shown in Palomero-Gallagher et al*.* (2020).

We additionally propose that hippocampal fields, as well as the dentate gyrus, are characterized by specific patterns of protein expression revealed by PCP4, ChrA, Rph3a and RGS-14 in the human brain. Supplementary Tables 3 and 4 summarize the relevant immunohistochemical identification features of the hippocampus and dentate gyrus.

In addition to regional protein expression patterns, this work shows that gross anatomical landmarks, apart from the fimbria, can help identify hippocampal fields. For instance, the apex of the *uncus* contains uCA3 and the Band of Giacomini, the hippocampal digitations all contain a dentate gyrus together with diverse hippocampal fields depending on the anteroposterior location, and the anterior pole of the gyrus *fasciolaris* is composed by fCA2 dorsally and fCA3 ventrally (see below, Results, and Supplementary Fig. 4b). Insausti and Amaral (2004) also noted that the gyrus *fasciolaris* contains the posterior end of CA3.

The posterior hippocampus is a particularly contentious region. In coronal sections, the posterior hippocampus has a very complicated appearance (Figs. 12, 13, 14, 15, 16; Supplementary Fig. 4). Adler et al. (2018) have suggested that adapting the cutting plane to the rotation of the posterior hippocampus may produce sections closely resembling the hippocampal body. However, this resemblance disappears in its terminal area, which lays ventral to the splenium of the corpus callosum (see supplementary material in Adler et al. 2018). The present immunohistochemical patterns support this idea: the gyrus *fasciolaris* and the dorsal hippocampus show notable deviations from the canonical immunostaining in the middle hippocampus (see RGS-14 and Rph3a in Table 8); by contrast, the dorsal posterior and ventral posterior fields follow an almost canonical appearance (with some exceptions for dpCA3). An additional complication of the posterior hippocampus are the Andreas Retzius gyri, whose number varies between individuals, and require examination in planes other than the coronal one (Adler et al. 2018; Palomero-Gallagher et al. 2020).

### CA2 boundaries and its overlap with CA3

Hippocampal CA2 is the most difficult field to identify reliably. CA2 neighbors the fimbrial insertion along the entire hippocampus: CA2 is anterior to the fimbria in the anterior hippocampus, is inferior to the fimbria in the middle hippocampus, and is posterior to the fimbria in the posterior hippocampus. In the anterior hippocampus, there are interindividual variations in the order of appearance of hippocampal fields in neuroimaging (de Flores et al. 2020); these may have to do with the presence of a minute vertical CA2 in the uncus as well as with interindividual variation in the volume of CA2 itself.

Despite the topographical relations of CA2 with the fimbria, its borders are difficult to trace and are, in fact, controversial (Hirama et al. 1997; Duvernoy 2005). While the medial limit of CA2 is traditionally set by the lateral end of mossy fibers from the dentate gyrus that target CA3 (Lorente de Nó, 1934), this criterion is now under debate especially in the rodent literature (Kohara et al. 2014; Fernandez-Lamo et al. 2019). The lateral limit of CA2 is less defined histologically, but ChrA has been shown to provide effective discrimination of the CA2/CA1 boundary in humans (Munoz 1990; current data); this boundary appears to be critically affected in mild cognitive impairment (Mueller et al. 2010). In non-human mammals the use of immunohistochemical or molecular markers for CA2 identification has challenged the definition of the CA3/CA2 boundary. In rodents, co-expression of RGS-14 and STEP in pyramidal cells has been proposed as criterion for CA2 identity (Dudek et al. 2016; Bienkowski et al. 2018). Moreover, it has been shown that dentate gyrus cells are able to form *functional* connections with ‘molecularly defined’ CA2 pyramidal cells (Kohara et al. 2014; Fernandez-Lamo et al. 2019). In addition, the mossy fiber pathway reaches the medial limit of CA1 in cats and other mammals (Hirama et al. 1997). This ambiguity hinders the topographical allocation of CA2-related functional features, like the lack of long-term potentiation (LTP) at glutamatergic synapses in the *stratum radiatum* (Zhao et al. 2005; Chevaleyre and Siegelbaum 2010).

In this work, we show that ChrA and RGS-14 stain pyramidal cells of CA2 allowing subdivision of CA2 into three distinct subfields termed CA2a-b-c from lateral to medial (following Munoz 1990), which is equivalent to the distal-to-proximal organization described in rodents (Fernandez-Lamo et al. 2019). The more medial ChrA + /RGS-14 + cells (CA2c pyramidal cells) are situated in a territory showing PCP4 + /Rph3a + mossy fibers which would otherwise qualify as CA3a and CA3b. These observations reinforce the idea of an identity between human medial CA2 (CA2c) and lateral CA3 (CA3a) which has been also demonstrated in functional studies in rodents (Zhang and Hernández 2012). We propose reserving the nomenclature CA3a for this overlapping territory (see also Ding and Van Hoesen 2015).

### Morpho-functional correlation

In this work, we describe a set of immunohistochemical features of a critical region between CA3 and CA2 that allow a precise distinction between subfields. Among all proteins used for this work, PCP4, Rph3a and RGS-14 are particularly involved in synaptic plasticity.

One of the main features of CA2 is its resistance to LTP (Dudek et al. 2016). Increased extracellular calcium levels (Simons et al. 2009) are known to elicit LTP in CA2 and PCP4 has been implicated in calcium buffering, providing a physiological role for PCP4 expression in CA2 pyramidal cells (Kleerekoper and Putkey 2009). However, we have not found PCP4 in human CA2 cells, a result that replicates previous findings (Renelt et al. 2014). This finding does not preclude a potential homologous role in human CA2, as PCP4 is present both in CA3a/CA2c and CA2a,b *strata radiata* (see Supplementary Fig. 5), regions taking part in networks where LTP has been described, and intracellular dynamics of PCP4 are still not fully understood (Hamada et al. 2014) PCP4 promotes conformational changes in cell membranes and the extension of filopodia-like projections involved in synaptic plasticity in dendritic spines (Yoshimura et al. 2016).

Our results show an overlapping distribution of PCP4 and Rph3a in the human hippocampus. Rph3a, conjointly with Rab3a, is present in the presynaptic apparatus, participating in the repletion, docking and fusion of vesicles (Tsuboi and Fukuda 2006; Lv et al. 2022). It is also known to be present at the postsynaptic level, clustering with GluN2A/PSD-95 (Stanic et al. 2015; Franchini et al. 2019), where it is required for the stabilization of GluN2A at synapses following LTP. Furthermore, the absence of Rph3a prevents modifications in spine density (Franchini et al. 2019, 2022). The combined presence of Rph3a + , PCP4 + neuropil in the *stratum lacunosum moleculare* and the CA3 *stratum lucidum*, as well as the CA2 *stratum radiatum*, may indicate a synergistic function in structural changes related to LTP in dendritic spines. We also find strong immunoreactivity for RGS-14, another protein related to LTP resistance in CA2, where it is known to block LTP by specifically reducing postsynaptic calcium levels and regulating the structural plasticity of dendritic spines (Lee et al. 2010; Evans et al. 2018). Further ultrastructural studies regarding the subcellular distribution of these proteins are required to understand the biological meaning of their expression patterns.

ChrA also proved to be useful in hippocampal parcellation. ChrA was described in the adrenal gland and its primary function is to concentrate catecholamines in vesicles of secretory cells (Hillarp 1959; Banks and Helle 1965), both in the peripheral nervous tissue and the brain (Taupenot et al. 2003; Helle 2010; Zhao et al. 2009; Montero-Hadjadje et al. 2009, reviewed by Machado et al. 2010). ChrA immunoreactivity has been described in the human hippocampus, and it was proposed to be one of the underlying factors of CA2 resistance to epilepsy-induced damage (Munoz 1990). The pattern of ChrA expression here described matches the one described using various antibodies including the LK2H10 (Munoz 1990) and WE-14 (Schafer et al. 2010). Differences in interneuron ChrA staining patterns in CA2 and CA3 between humans and other mammals might be explained by differences in the antibody batch (Schafer et al. 2010).

Sarnat (2013) attributed ChrA immunoreactivity in CA3 neuropil to terminal axons of CA2. However, the spotted immunoreactivity present in axon terminals in CA3a/CA2c may also correspond to projections arising from the *locus coeruleus* that are known to specifically signal to this region, being involved in novelty-specific memory enhancement (Takeuchi et al. 2016; McNamara and Dupret 2017; Wagatsuma et al. 2018). Extrahippocampal catecholaminergic projections to CA3, both arising from the *locus coeruleus* and ventral tegmental area can induce changes within the CA3 microcircuits after single trials (Wagatsuma et al. 2018), being potent drivers of synaptic plasticity (Lisman and Grace 2005). Dopamine innervation to the hippocampus, arising in *substantia nigra pars compacta* and the ventral tegmental area, has been also shown to play a role in goal encoding and path selection in mice (Retailleau et al. 2013).

Catecholaminergic signaling in the hippocampus has been shown to elicit long-lasting LTP in the hippocampus through the β-adrenoceptors (Tenorio et al. 2010). Granins have been implicated in dendrite outgrowth as well as the maintenance of cell morphology in immature populations (Chen, et al. 1992; Zhang et al. 2020). The paracrine role of ChrA and ChrA-derived peptides and its calcium-binding properties may explain the cytoplasmic expression in CA2 (D’amico et al. 2014). Further studies are needed to elucidate whether CA2 constitutes the origin of CA3a,b/CA2c ChrA + terminals. ChrA + terminals are also present in the hilar region close to the dentate gyrus subgranular zone, where catecholaminergic signaling is also known to promote hippocampal neurogenesis via β_3_-adrenoceptors (Jhaveri et al. 2010).

## Conclusions

A combination of immunohistochemical markers (Rph3a, PCP4, Chromogranin A, RGS-14) allows precise differentiation of fields and subfields within the human hippocampus proper.

We found major regional histochemical differences within hippocampal fields, particularly in the head and tail of the human hippocampus. Also, there is some overlap between medial CA2 (CA2c) and lateral CA3 (CA3a) in the human hippocampus. The regional patterns of protein expression differ along the longitudinal axis of the human hippocampus from those described in other mammals. The maps and the topographical references provided in the Figures and Tables of this article will help neuroradiologists and pathologists to identify fields and landmarks in the human hippocampus.

### Supplementary Information

Below is the link to the electronic supplementary material.

Supplementary file1 (TIF 11652 KB)—Fig. 1: Confocal microscopy images showing the distribution of Chromogranin A, parvalbumin, RGS-14, and Rph3a at cellular level in the human hippocampus. a-c: Chromogranin A is expressed in pyramidal neurons (arrows), but not interneurons (asterisks) along lateral CA3. d-f: In CA2 pyramidal layer, Chromogranin A shows widespread co-expression with RGS-14 in pyramidal cells. g-l: Rph3a is expressed in fibers and terminals across the hilus (arrows in k). Cytoplasmic expression is restricted to Parvalbumin+ interneurons of the subgranular zone (see g-i). The labels in the left lower angles indicate the brain corresponding to each picture (see Supplementary Table 1). Abbreviations: DGgl, dentate gyrus granule cell layer; -pcl as a suffix, pyramidal cell layer; sgz, subgranular zone.

Supplementary file2 (TIF 14257 KB)—Fig. 2: Continuity of CA3 in the perifimbrial hippocampus near the uncal apex. The uncal portion of CA3 reaches the hilar region of the vertical hippocampus from lateral to medial. The vertical dentate gyrus is, at this point, inverted. The posterior concavity of the dentate gyrus at this level is the uncal apex (UA in b). Abbreviations: -h as a suffix, hilar; iBG, inferior Band of Giacomini; Sb, subiculum; u- as a prefix, uncal; v- as a prefix, vertical; I, dentate gyrus of digitation #1. Red broken line: hippocampal fissure.

Supplementary file3 (TIF 16104 KB)—Fig. 3: 3-D reconstruction of the anterior hippocampal region (generated by FreeD software, Andrey and Maurin, 2005). For the sake of clarity, the light blue rectangles in g and h indicate the plane nearest to the reader (anterior plane in g, posterior plane in h). #1, #2, #3, #4: hippocampal digitations; I, II, II: dentate gyrus of hippocampal digitations #1, #2, and #3, respectively.

Supplementary file4 (TIF 9040 KB)—Fig. 4: Anteroposterior organization of the gyrus fasciolaris (GF), the fasciola cinerea (fc), and the dorsal hippocampus. Note also the dorsalmost part of the posterior hippocampus and dentate gyrus (#5) and the gyri of Andreas Retzius (lateral #6 and medial #7). The dentatofasciolar sulcus (dfs in c, d, e) is a medial expansion of the inner blade of the posterior hippocampal fissure (** in c, d, e) near the point where it becomes superficial (*** in c). The fasciola cinerea, which is a rudimentary dentate gyrus that also presents a rudimentary medial blade (**** in d and e), is organized around the dfs. Note the presence of a vestigial dorsal or fasciolar fimbriodentate junction (*) attached to the ventral surface of the corpus callosum (CC). Abbreviations: al, alveus; d- as a prefix, dorsal; dp as a prefix, dorsal posterior; f- as a prefix, fasciolar; scs, subcallosal sulcus. Red broken line: hippocampal fissure.

Supplementary file5 (TIF 10484 KB)—Fig. 5: Main immunohistochemical features of hippocampal CA2a,b (lateral CA2). The stratum lacunosum moleculare, which mainly contains synapses between pyramidal cells and fibres arising from the periarchicortex (C), shows dense PCP4+, Rph3a+ neuropil. The stratum radiatum forms closer to the pyramidal cell soma and its main feature is the presence of a faintly neuropil for both proteins. The stratum radiatum contains in CA2 projections either from Schaffer collaterals or from the entorhinal cortex. The whole pyramidal neuron shows RGS-14 immunoreactivity, while its soma shows punctate cytoplasmic expression of Chromogranin A. Abbreviations: EC, entorhinal cortex, ScC, Schaffer collaterals.

Supplementary file6 (PDF 94 KB)—Table 1: Summary of cases.

Supplementary file7 (PDF 192 KB)—Table 2: Battery of primary antibodies used in this work

Supplementary file8 (PDF 81 KB)—Table 3: Main immunohistochemical features of the human dentate gyrus.

Supplementary file9 (PDF 124 KB)—Table 4: Main immunohistochemical features of the human middle hippocampus.

Supplementary file10 (PDF 107 KB)—Table 5: Coordinates of the main macro- and micro-anatomical landmarks in the anterior hippocampus.

Supplementary file11 (PDF 111 KB)—Table 6: Main immunohistochemical features of hippocampal fields specific to the anterior hippocampus.

Supplementary file12 (PDF 90 KB)—Table 7: Coordinates of the main macro and microanatomical landmarks in the posterior hippocampus.

Supplementary file13 (PDF 114 KB)—Table 8: Main immunohistochemical features of modified hippocampal fields in the posterior hippocampus.

Supplementary file14 (PDF 125 KB)—Table 9: Field distribution along the hippocampal longitudinal (postero-anterior) axis

## Data Availability

All the data presented in the manuscript can be requested to the authors.
