# Supplementary figures and images for: Immunohistochemical field parcellation of the human hippocampus along its antero-posterior axis
Source: Brain Struct Funct. 2024 Jan 5;229(2):359–85. doi: 10.1007/s00429-023-02725-9 (PMC10917878; doi:10.1007/s00429-023-02725-9)

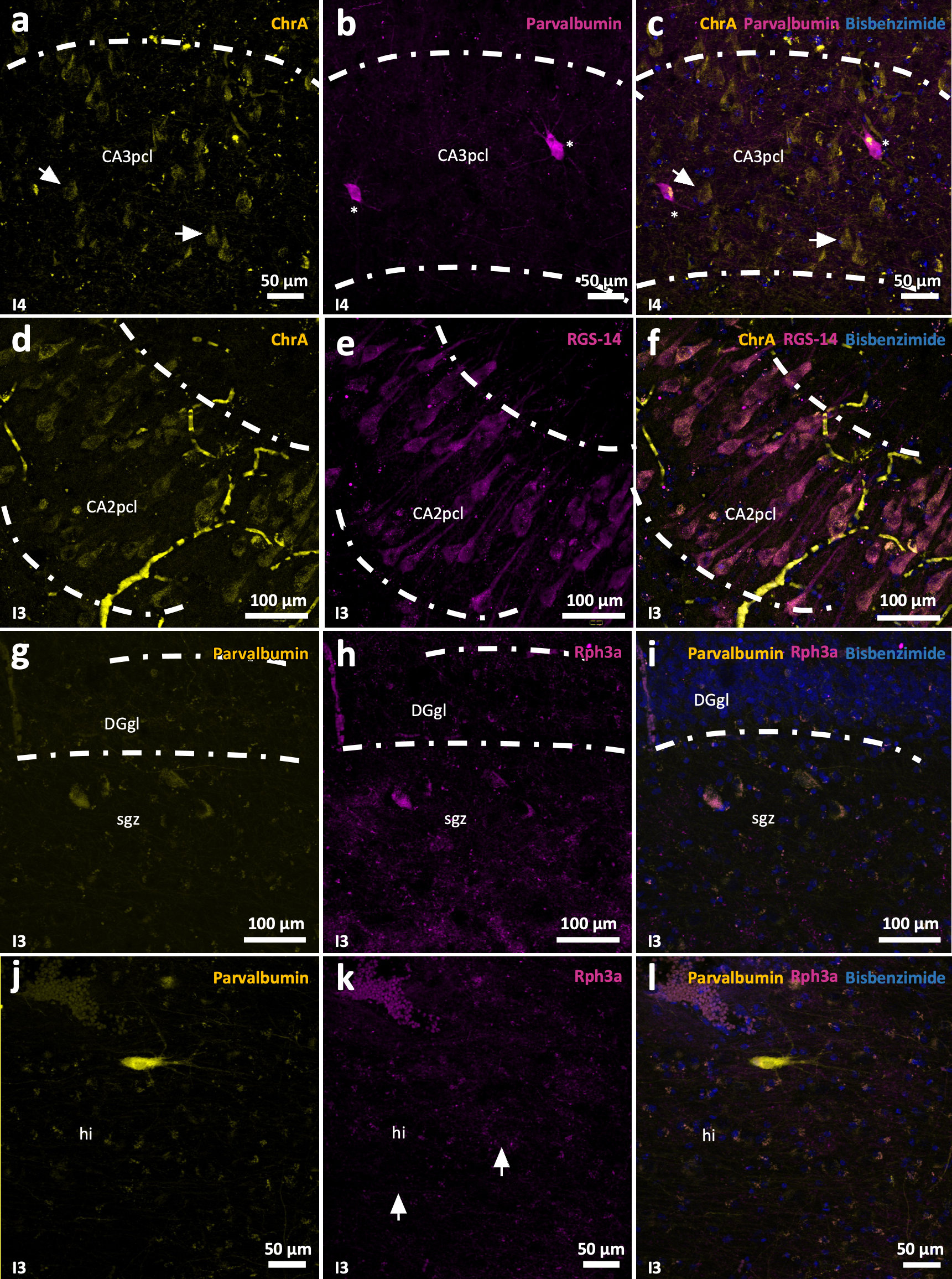

Supplement: Supplementary file 1 — Supplementary file1 (TIF 11652 KB)—Fig. 1: Confocal microscopy images showing the distribution of Chromogranin A, parvalbumin, RGS-14, and Rph3a at cellular level in the human hippocampus. a-c: Chromogranin A is expressed in pyramidal neurons (arrows), but not interneurons (asterisks) along lateral CA3. d-f: In CA2 pyramidal layer, Chromogranin A shows widespread co-expression with RGS-14 in pyramidal cells. g-l: Rph3a is expressed in fibers and terminals across the hilus (arrows in k). Cytoplasmic expression is restricted to Parvalbumin+ interneurons of the subgranular zone (see g-i). The labels in the left lower angles indicate the brain corresponding to each picture (see Supplementary Table 1). Abbreviations: DGgl, dentate gyrus granule cell layer; -pcl as a suffix, pyramidal cell layer; sgz, subgranular zone. [file 429_2023_2725_MOESM1_ESM.tif]

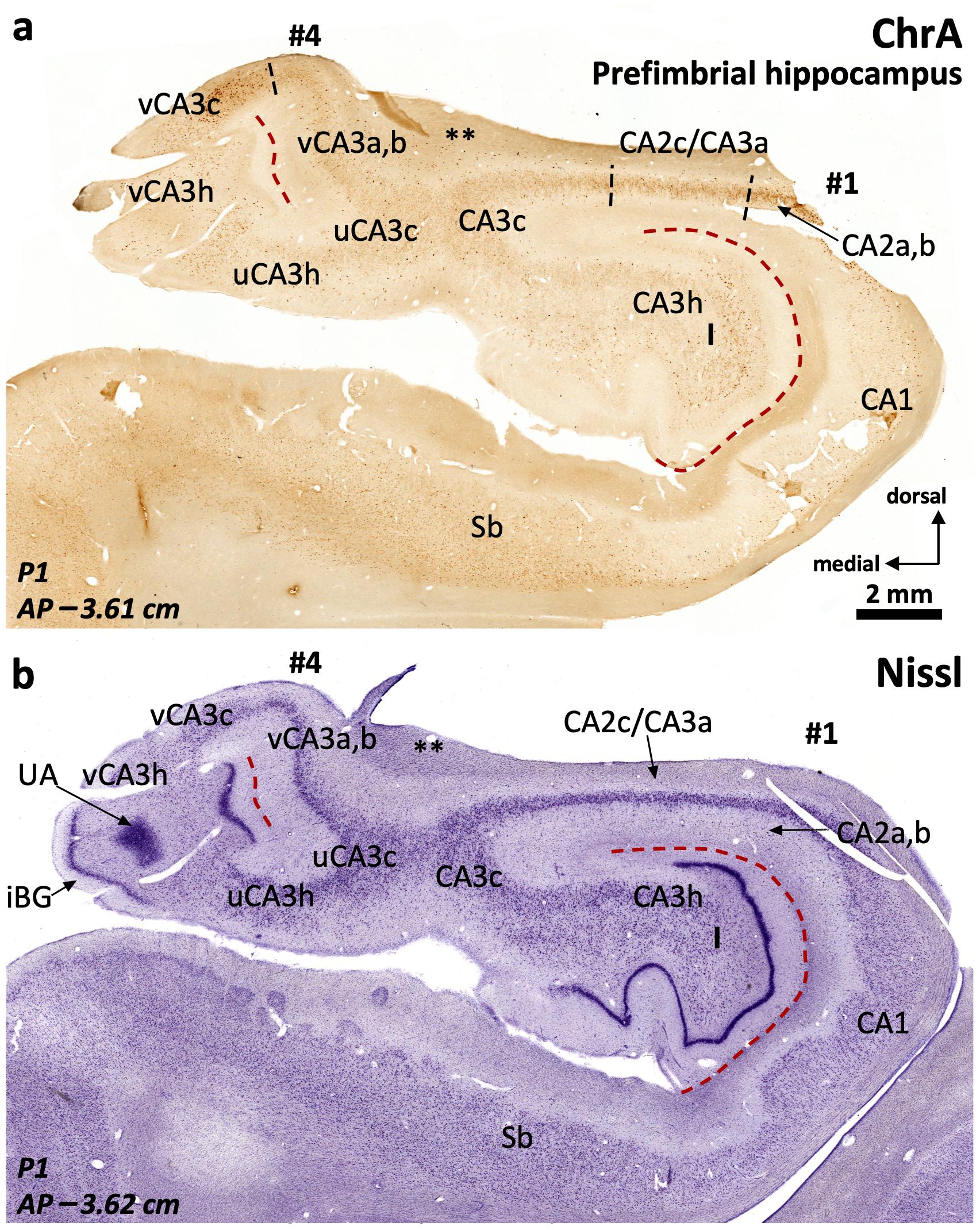

Supplement: Supplementary file 2 — Supplementary file2 (TIF 14257 KB)—Fig. 2: Continuity of CA3 in the perifimbrial hippocampus near the uncal apex. The uncal portion of CA3 reaches the hilar region of the vertical hippocampus from lateral to medial. The vertical dentate gyrus is, at this point, inverted. The posterior concavity of the dentate gyrus at this level is the uncal apex (UA in b). Abbreviations: -h as a suffix, hilar; iBG, inferior Band of Giacomini; Sb, subiculum; u- as a prefix, uncal; v- as a prefix, vertical; I, dentate gyrus of digitation #1. Red broken line: hippocampal fissure. [file 429_2023_2725_MOESM2_ESM.tif]

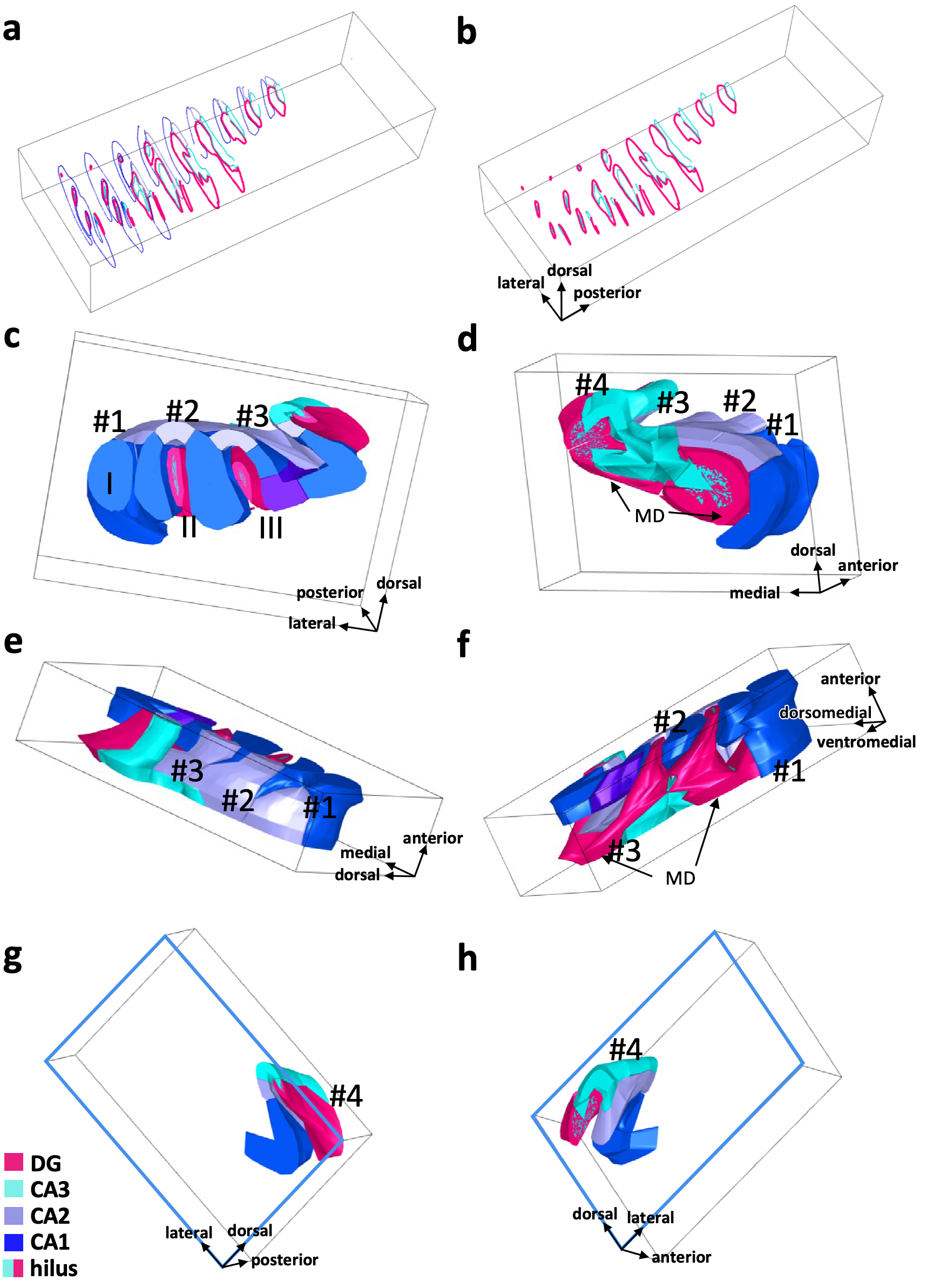

Supplement: Supplementary file 3 — Supplementary file3 (TIF 16104 KB)—Fig. 3: 3-D reconstruction of the anterior hippocampal region (generated by FreeD software, Andrey and Maurin, 2005). For the sake of clarity, the light blue rectangles in g and h indicate the plane nearest to the reader (anterior plane in g, posterior plane in h). #1, #2, #3, #4: hippocampal digitations; I, II, II: dentate gyrus of hippocampal digitations #1, #2, and #3, respectively. [file 429_2023_2725_MOESM3_ESM.tif]

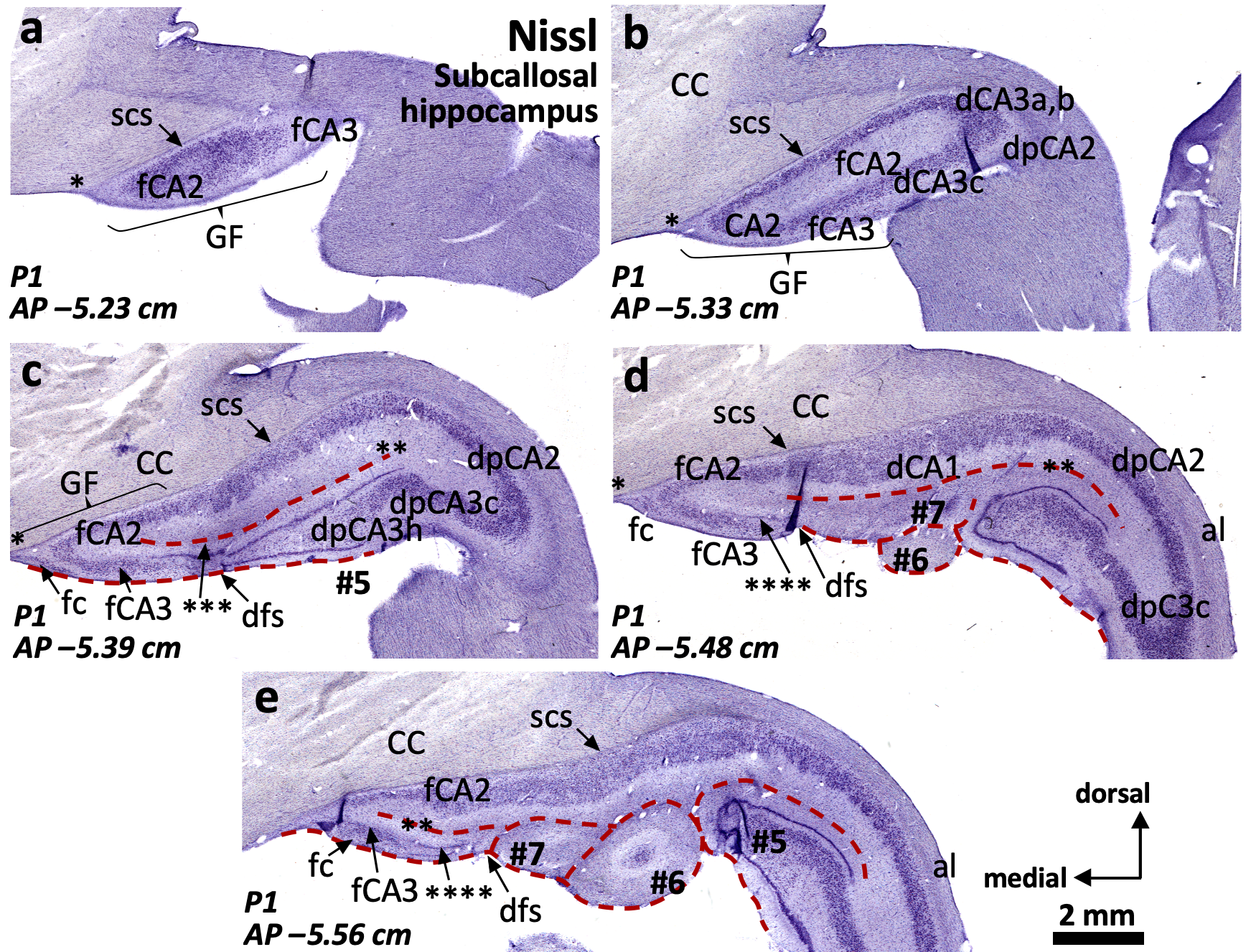

Supplement: Supplementary file 4 — Supplementary file4 (TIF 9040 KB)—Fig. 4: Anteroposterior organization of the gyrus fasciolaris (GF), the fasciola cinerea (fc), and the dorsal hippocampus. Note also the dorsalmost part of the posterior hippocampus and dentate gyrus (#5) and the gyri of Andreas Retzius (lateral #6 and medial #7). The dentatofasciolar sulcus (dfs in c, d, e) is a medial expansion of the inner blade of the posterior hippocampal fissure (** in c, d, e) near the point where it becomes superficial (*** in c). The fasciola cinerea, which is a rudimentary dentate gyrus that also presents a rudimentary medial blade (**** in d and e), is organized around the dfs. Note the presence of a vestigial dorsal or fasciolar fimbriodentate junction (*) attached to the ventral surface of the corpus callosum (CC). Abbreviations: al, alveus; d- as a prefix, dorsal; dp as a prefix, dorsal posterior; f- as a prefix, fasciolar; scs, subcallosal sulcus. Red broken line: hippocampal fissure. [file 429_2023_2725_MOESM4_ESM.tif]

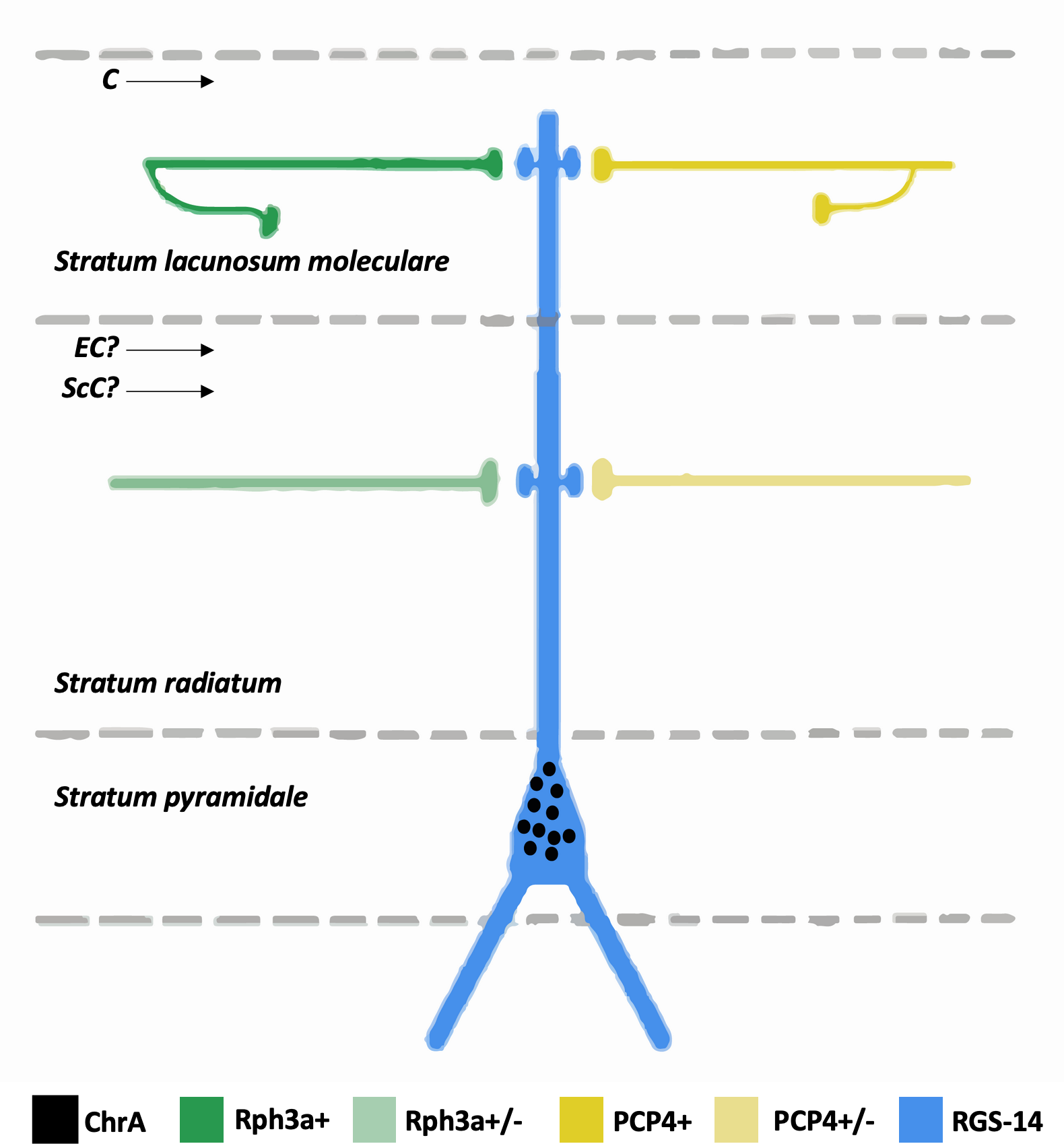

Supplement: Supplementary file 5 — Supplementary file5 (TIF 10484 KB)—Fig. 5: Main immunohistochemical features of hippocampal CA2a,b (lateral CA2). The stratum lacunosum moleculare, which mainly contains synapses between pyramidal cells and fibres arising from the periarchicortex (C), shows dense PCP4+, Rph3a+ neuropil. The stratum radiatum forms closer to the pyramidal cell soma and its main feature is the presence of a faintly neuropil for both proteins. The stratum radiatum contains in CA2 projections either from Schaffer collaterals or from the entorhinal cortex. The whole pyramidal neuron shows RGS-14 immunoreactivity, while its soma shows punctate cytoplasmic expression of Chromogranin A. Abbreviations: EC, entorhinal cortex, ScC, Schaffer collaterals. [file 429_2023_2725_MOESM5_ESM.tif]
