# Supplementary material for: Immunohistochemical field parcellation of the human hippocampus along its antero-posterior axis
Source: Brain Struct Funct. 2024 Jan 5;229(2):359–85. doi: 10.1007/s00429-023-02725-9 (PMC10917878; doi:10.1007/s00429-023-02725-9)
Supplement: Supplementary file 6 — Supplementary file6 (PDF 94 KB)—Table 1: Summary of cases. [file 429_2023_2725_MOESM6_ESM.pdf]

Supplementary Table 1: Summary of cases.

Case	*Age*	*Sex*	*Cause of death*	*Post-mortem delay (h)*	*Fixation method*	*Paraffin embedding*	*Sectioning*
**P1**	58	Male	Polycythemia Vera	2,5	Trans-carotid and basilar perfusion	No	50 µm sections in parallel series, stereotaxic slicing
**P2**	67	Male	Septicemia	3,5	Trans-carotid and basilar perfusion	No	50 µm sections in parallel series, stereotaxic slicing
**I1**	58	Female	Pneumonia	4,5	Immersion	No	50 µm consecutive sections, no stereotaxic slicing
**I2**	29	Male	Subdural bleeding	4	Immersion	No	50 µm consecutive sections, no stereotaxic slicing
**I3**	32	Male	Enterocolitis	5	Immersion	No	50 µm consecutive sections, no stereotaxic slicing
**I4**	68	Male	Dilated cardiomyopathy	16	Immersion	No	50 µm consecutive sections, no stereotaxic slicing
**I5**	71	Male	Metastatic disease of unknown origin	5	Immersion	No	50 µm consecutive sections, no stereotaxic slicing
**Pf1**	42	Male	Unknown	4,5	Immersion	Yes	20 µm selected sections, no stereotaxic slicing
**Pf2**	60	Female	Unknown	11,5	Immersion	Yes	20 µm selected sections, no stereotaxic slicing
**Pf3**	52	Female	Unknown	5,5	Immersion	Yes	20 µm selected sections, no stereotaxic slicing
**Pf4**	52	Male	Unknown	4,5	Immersion	Yes	20 µm selected sections, no stereotaxic slicing
**F19**	65	Male	Metastatic disease of unknown origin	4	Trans-femoral perfusion	No	Macroscopic exam
