# Supplementary material for: Immunohistochemical field parcellation of the human hippocampus along its antero-posterior axis
Source: Brain Struct Funct. 2024 Jan 5;229(2):359–85. doi: 10.1007/s00429-023-02725-9 (PMC10917878; doi:10.1007/s00429-023-02725-9)
Supplement: Supplementary file 7 — Supplementary file7 (PDF 192 KB)—Table 2: Battery of primary antibodies used in this work [file 429_2023_2725_MOESM7_ESM.pdf]

Supplementary Table 2: Battery of primary antibodies used in this work

Protein	***Antibody*** *(Reference & dilution)*	***Expression pattern*** *(Murine hippocampus)*	***Expression pattern*** *(Human hippocampus)*
Astn 2	anti-Astn2 ab121906 rabbit polyclonal 1/10 – 1/50	mRNA differential expression along longitudinal axis[1]	Not found
Nr3c2	anti- mineralocorticoid receptor, Nr3c2 mouse monoclonal H10E4C9F 1/500	mRNA differential expression along longitudinal axis[1]	Dense fiber plexus in Deep pyramidal layers / *stratum oriens.* No pattern overall.
Wfs-1	anti-Wfs1 rabbit polyclonal PA5-6064 1/1750	mRNA expression in dorsal murine hippocampus[1]	Strong expression in non-pyramidal hiliar cells in both hippocampal body and posterior hippocampus. Dense fibrillar plexus around cellular layers of posterior hippocampus
Coll11A1	anti-Col11A1 rabbit polyclonal LS-C352032 1/100	mRNA differential expression along longitudinal axis and different *laminae*[2]	Scattered hiliar cells Scattered fibers in pyramidal layers Subicular pyramidal cells. No pattern overall.
Neurotensin	anti-neurotensin mouse monoclonal SAB4200703 1/500	Subicular layers 1-3 in dorsal murine hippocampus[1,2]	Subicular pyramidal cells, with no laminar or longitudinal pattern
Dlk1	anti-Dlk1 mouse monoclonal 3A10 1/100- 1/200	Subicular layer 2[2]	Not found
Coch1	anti-Coch1 mouse monoclonal ab195959 1/50-1/200	mRNA differential expression along longitudinal axis and different *laminae*[1,2]	Scattered non-pyramidal cells with no identifiable pattern
Dclk3	anti-Dclk3 ab113367 rabbit polyclonal 1/150	Pyramidal cells in dorsal CA1 fields[2]	Widespread cytoplasmic reactivity in pyramidal cells, stronger in CA3 cells. No identifiable pattern
Rph3a	**anti-Rph3a rabbit polyclonal HPA002475 Sigma-Aldrich® 1/500**	**Laminar patterns in ventral pyramidal layers**[2]	**See Results**
Amigo1	anti-Amigo1 rabbit polyclonal HPA046152 1/1000	CA2[1]	Widespread nuclear staining of pyramidal cells, with neither field, nor laminar specificity.
Itga7	anti-Itga7 r mouse monoclonal ab195959 1/500	Laminar pattern in pyramidal cell layers[1,2]	Scattered fibers with no pattern
PCP4	**anti-PCP4 rabbit polyclonal HPA005792 Sigma-Aldrich® 1/750**	**CA2 and subicular pyramidal cells**[3]	**See Results**
ChrA	**Mouse monoclonal LK2H10 Cell Marque® 1/100**	**CA (mostly CA3) pyramidal neurons, Neuropil * (WE-14, see discussion)**[4]	**See Results**
RGS-14	**Anti-RGS-14 Rabbit polyclonal 16258-1-AP Proteintech 1/150**	**CA2 and *fasciola cinerea*, CA1 (sparse) ***[5]	**See Results**
Parvalbumin	Anti-Parvalbumin mouse monoclonal PV235 1/400 Anti-Parvalbumin rabbit polycolonal PV27 1/400	**	See Results & Supplementary Figure 1

Supplementary Table 2 Legend: Bold indicates patterned expression in human hippocampus. * indicates the utilization of a different batch compared to the one in which the mentioned pattern was described. **: antibodies used only in immunofluorescence experiments in selected sections, not for regionalization (1: Fanselow and Dong, 2010; 2: Bienkowski et al, 2018; 3: Renelt et al 2014; 4: Munoz, 1990; 5: Evans et al, 2014).
