# Supplementary material for: Immunohistochemical field parcellation of the human hippocampus along its antero-posterior axis
Source: Brain Struct Funct. 2024 Jan 5;229(2):359–85. doi: 10.1007/s00429-023-02725-9 (PMC10917878; doi:10.1007/s00429-023-02725-9)
Supplement: Supplementary file 8 — Supplementary file8 (PDF 81 KB)—Table 3: Main immunohistochemical features of the human dentate gyrus. [file 429_2023_2725_MOESM8_ESM.pdf]

Supplementary Table 3: Main immunohistochemical features of the human dentate gyrus.

Marker	***Layer***		
	Molecular layer	***Granule cell layer***	***Hilus***
**PCP4**	Moderate neuropil staining	Cytoplasmic staining in granule cells	Light neuropil staining
**Rph3a**	Dense neuropil staining	No staining	Scattered interneurons
**ChrA**	No staining	No staining	No staining
**RGS-14**	Diffuse light neuropil staining	Diffuse light neuropil staining	Diffuse light neuropil staining
