# Supplementary material for: Immunohistochemical field parcellation of the human hippocampus along its antero-posterior axis
Source: Brain Struct Funct. 2024 Jan 5;229(2):359–85. doi: 10.1007/s00429-023-02725-9 (PMC10917878; doi:10.1007/s00429-023-02725-9)
Supplement: Supplementary file 9 — Supplementary file9 (PDF 124 KB)—Table 4: Main immunohistochemical features of the human middle hippocampus. [file 429_2023_2725_MOESM9_ESM.pdf]

Supplementary Table 4: Main immunohistochemical features of the human middle hippocampus.

Field	*Marker*	*Layer*			
		**Molecular layer/*stratum lacunosum moleculare***	***Stratum radiatum***	**Pyramidal cell layer**	**Plexiform layer/ *stratum oriens***
**CA3h**	PCP4	Dense neuropil staining			
	Rph3a	PV+ and PV- interneurons, dense neuropil staining, and ubiquitous terminals			
	ChrA	Ubiquitous staining in terminals and scant, scattered neurons			
	RGS-14	Diffuse light neuropil staining			
**CA3c**	PCP4	No staining		No staining in pyramidal cells. Light neuropil staining	Light neuropil staining
	Rph3a	Light neuropil staining		No staining in pyramidal cells. Light neuropil staining	Light neuropil staining
	ChrA	No staining		Neuropil and terminals	No staining
	RGS-14	Light neuropil staining		Moderate to dense neuropil staining, no pyramidal cell staining	Light diffuse neuropil staining
**CA3 a,b**	PCP-4	Light neuropil staining (superficial) No staining (deep)		No staining in pyramidal cells. Light neuropil staining. Strong staining in deep and superficial mossy fibers.	No staining
	Rph3a	Moderate neuropil staining (superficial) Light neuropil staining (deep)		No staining in pyramidal cells. Light neuropil staining. Strong staining in deep and superficial mossy fibers.	Light neuropil staining
	ChrA	No staining		Cytoplasmic staining in pyramidal cells. Scattered terminals.	No staining
	RGS-14	Subtle neuropil staining		Strong cytoplasmic staining in pyramidal cells (probably restricted to CA3a, see discussion)	Light neuropil staining
**CA2**	PCP4	Light neuropil staining	Subtle neuropil staining	No staining	No staining
	Rph3a	Moderate neuropil staining	Light neuropil staining (*)	Light neuropil staining (*)	No staining
	ChrA	No staining	No staining	Cytoplasmic staining in pyramidal cells	No staining
	RGS-14	Moderate neuropil staining / Strong neuropil staining in CA2b	Strong staining in basal segment of apical dendrites of pyramidal cells	Strong cytoplasmic staining in pyramidal cells	Light neuropil staining
**CA1**	PCP4	Light staining	No staining	No staining	Scattered neurons, mostly ventromedially
	Rph3a	Strong neuropil staining	Moderate neuropil staining	Moderate neuropil staining	No staining
	ChrA	No staining (lateral-superficial) Light staining (medial and lateral-deep)	Light neuropil staining	No staining	Scattered neurons
	RGS-14	No staining (lateral-superficial) Moderate neuropil staining (medial)	Subtle neuropil staining	Strong neuropil staining	Light neuropil staining
**Subiculum**	PCP4	No neuropil staining. Apical processes of deep pyramidal cells ramifying in lateral subiculum		Cytoplasmic staining in deep pyramidal cells	Scattered neurons
	Rph3a	No staining		Light neuropil staining	Light neuropil staining
	ChrA	No staining		Scattered neurons in deep layers	Scattered neurons
	RGS-14	No staining (superficial) Light neuropil staining (deep)		Strong-to-moderate neuropil staining	Light neuropil staining
