# Supplementary material for: Immunohistochemical field parcellation of the human hippocampus along its antero-posterior axis
Source: Brain Struct Funct. 2024 Jan 5;229(2):359–85. doi: 10.1007/s00429-023-02725-9 (PMC10917878; doi:10.1007/s00429-023-02725-9)
Supplement: Supplementary file 10 — Supplementary file10 (PDF 107 KB)—Table 5: Coordinates of the main macro- and micro-anatomical landmarks in the anterior hippocampus. [file 429_2023_2725_MOESM10_ESM.pdf]

Supplementary Table 5: Coordinates of the main macro- and micro-anatomical landmarks in the anterior hippocampus.

Hippocampal region	*Main anatomical landmarks*	*P1 AP (cm)*	*P2 AP (cm)*
**Anterior pole**		-2,94	-3,18
	Most anterior hippocampal digitations (II,III)	-2,97	-3,25
**Prefimbrial hippocampus**	Dentate gyrus granule cell layer in II, III	-3,07	-3,32
	Anterior end of digitation I	-3,2	-3,3
	Anterior limit of CA2	-3,2	-3,4
	Granule cell layer in I	-3,4	-3,48
	Posterior end of uSC	-3,45	-3,57
	Posterior end of uCA1	-3,5	-3,63
	Anterior end of vertical hippocampus	*Circa* -3	-3,08
	Continuity of CA2 as a single rim	*Circa* -3,34	-3,5
**Perifimbrial hippocampus**	Anterior end of uCA3 a,b and CA3 a,b	-3,47	*Circa* -3,70
	Unification of the Band of Giacomini	-3,58	-3,78
	Continuity of CA3 as a single rim	-3,64	-3,78
	Continuity of the Hippocampal fissure	-3,65	-3,8
	Uncal apex	-3,7	-3,9
