# Supplementary material for: Immunohistochemical field parcellation of the human hippocampus along its antero-posterior axis
Source: Brain Struct Funct. 2024 Jan 5;229(2):359–85. doi: 10.1007/s00429-023-02725-9 (PMC10917878; doi:10.1007/s00429-023-02725-9)
Supplement: Supplementary file 11 — Supplementary file11 (PDF 111 KB)—Table 6: Main immunohistochemical features of hippocampal fields specific to the anterior hippocampus. [file 429_2023_2725_MOESM11_ESM.pdf]

Supplementary Table 6: Main immunohistochemical features of hippocampal fields specific to the anterior hippocampus.

Field	***Marker***	***Layer***		
		**Molecular layer/*stratum lacunosum moleculare***	**Pyramidal cell layer**	**Plexiform layer/ *stratum oriens***
**vCA3c**	PCP4	No staining	No staining	Not identifiable
	Rph3a	No staining	No staining	Not identifiable
	ChrA	No staining	Strong cytoplasmic staining, strong neuropil staining, ubiquitous terminals	Not identifiable
	RGS-14	Light neuropil staining	Moderate neuropil staining, scattered terminals	Light neuropil staining
**vCA3 a,b**	PCP-4	Light neuropil staining (superficial)<br>No staining (deep)	No staining in pyramidal cells. Light neuropil staining. Strong staining in deep and superficial mossy fibers.	No staining
	Rph3a	Moderate neuropil staining	Light neuropil staining	No staining
	ChrA	No staining	Light neuropil staining. Scattered terminals.	No staining
	RGS-14	Subtle neuropil staining	Moderate neuropil staining	Light neuropil staining
**vCA2**	PCP4	No staining	No staining	No staining
	Rph3a	Moderate neuropil staining (superficial)<br>Light neuropil staining (deep)	Light neuropil staining (*)	No staining
	ChrA	No staining (superficial)<br>Moderate neuropil staining	Cytoplasmic staining in pyramidal cells. Scattered terminals. Moderate neuropil staining.	No staining
	RGS-14	Moderate neuropil staining	Strong cytoplasmic staining in pyramidal cells. Moderate neuropil staining	Light neuropil staining
**vCA1**	PCP4	Light neuropil staining	Cytoplasmic staining in deep pyramidal cells (progressively converging towards a superficial single PCP4- layer in vCA2)	No staining
	Rph3a	Moderate neuropil staining<br>Moderate neuropil staining (medial)	Light neuropil staining	No staining
	ChrA	Light neuropil staining (superficial)<br>No staining (deep)	Scattered non-pyramidal cells. Moderate neuropil staining	No staining
	RGS-14	Moderate neuropil staining	Moderate-to-strong neuropil staining	Light neuropil staining
**uCA1**	PCP4	Moderate neuropil staining with prominently positive dendritic arborizations of pyramidal cells	Strong cytoplasmic staining in deep pyramidal cells	No staining
	Rph3a	Strong diffuse neuropil staining	Moderate neuropil staining	Light neuropil staining
	ChrA	No staining	Scattered non-pyramidal neurons	No staining
	RGS-14	Light neuropil staining (superficial)<br>Moderate neuropil staining (intermediate)<br>Light neuropil staining (deep)	Strong-to-moderate neuropil staining	Light neuropil staining
