# Supplementary material for: Immunohistochemical field parcellation of the human hippocampus along its antero-posterior axis
Source: Brain Struct Funct. 2024 Jan 5;229(2):359–85. doi: 10.1007/s00429-023-02725-9 (PMC10917878; doi:10.1007/s00429-023-02725-9)
Supplement: Supplementary file 12 — Supplementary file12 (PDF 90 KB)—Table 7: Coordinates of the main macro and microanatomical landmarks in the posterior hippocampus. [file 429_2023_2725_MOESM12_ESM.pdf]

Supplementary Table 7: Coordinates of the main macro and microanatomical landmarks in the posterior hippocampus.

Main anatomical landmarks	***P1 AP (cm)***	***P2 AP (cm)***
Contact between gyrus *fasciolaris* and posterior hippocampus	-5,38	-5,45
Posterior end of the fimbria	-5,45	-5,38
Posterior end of CA3, continuity of posterior CA2	-5,86	-5,85
Posterior end of CA1	-5,7	-5,85
Posterior end of hippocampus (posterior subiculum)	-5,9	-6
