# Supplementary material for: Immunohistochemical field parcellation of the human hippocampus along its antero-posterior axis
Source: Brain Struct Funct. 2024 Jan 5;229(2):359–85. doi: 10.1007/s00429-023-02725-9 (PMC10917878; doi:10.1007/s00429-023-02725-9)
Supplement: Supplementary file 13 — Supplementary file13 (PDF 114 KB)—Table 8: Main immunohistochemical features of modified hippocampal fields in the posterior hippocampus. [file 429_2023_2725_MOESM13_ESM.pdf]

Supplementary Table 8: Main immunohistochemical features of modified hippocampal fields in the posterior hippocampus.

Marker		*Layer*		
		**Molecular layer/*stratum lacunosum moleculare***	**Pyramidal cell layer**	**Plexiform layer/ *stratum oriens***
**fCA3**	PCP4	No staining	Moderate neuropil staining.	Not identifiable
	Rph3a	No staining	Sparse fibers	Not identifiable
	ChrA	No staining	Strong cytoplasmic staining, moderate neuropil staining, ubiquitous terminals	Not identifiable
	RGS-14	No staining	Moderate neuropil staining, Deep pyramidal cells.	Not identifiable
**fCA2**	PCP-4	Light-to-moderate neuropil staining	No staining	No staining
	Rph3a	Moderate neuropil staining	Scattered fibers, terminations and neuropil throughout its whole depth	No staining
	ChrA	No staining	Strong cytoplasmic punctate staining in pyramidal cells	No staining
	RGS-14	Moderate neuropil staining	Strong neuropil staining	Strong neuropil staining
**dpCA3**	PCP4	No staining	No staining in pyramidal cells. Light neuropil staining	Light neuropil staining
	Rph3a	Light neuropil staining	No staining in pyramidal cells. Light neuropil staining	Light neuropil staining
	ChrA	No staining	Neuropil and terminals	No staining
	RGS-14	No staining	Moderate neuropil staining, cytoplasmic staining in deep pyramidal cells.	No staining
