# Supplementary material for: Immunohistochemical field parcellation of the human hippocampus along its antero-posterior axis
Source: Brain Struct Funct. 2024 Jan 5;229(2):359–85. doi: 10.1007/s00429-023-02725-9 (PMC10917878; doi:10.1007/s00429-023-02725-9)
Supplement: Supplementary file 14 — Supplementary file14 (PDF 125 KB)—Table 9: Field distribution along the hippocampal longitudinal (postero-anterior) axis [file 429_2023_2725_MOESM14_ESM.pdf]

Supplementary Table 9: Field distribution along the hippocampal longitudinal (postero-anterior) axis

Hippocampal region				***Hippocampal fields (+hippocampal fissure)***				
	Gyrus *fasciolaris*			Dentato-fasciolar sulcus	Fasciola cinerea	**fCA3**	**fCA2**	**dCA1**
Posterior hippocampus/ Hippocampal tail	*Posterior hippocampus*	Dorsal posterior hippocampus		Posterior hippocampal fissure	Posterior dentate gyrus including posterior *margo denticulatus*	**dpCA3**	dpCA2/pCA2	pCA1
		Ventral posterior hippocampus				vpCA3	vpCA2/pCA2	pCA1
Middle hippocampus/ Hippocampal body				Hippocampal fissure (outer Blade/Deep hippocampal fissure + inner Blade/superficial hippocampal fissure)	Dentate gyrus including *margo denticulatus*	CA3	CA2	CA1
Anterior hippocampus/ Hippocampal head	*Perifimbrial hippocampus*	#4 (medial digitation)	Vertical Hippocampus	Superficial hippocampal fissure	Inferior/lower Band of Giacomini	**vCA3**	**vCA2**	**vCA1**
			Uncus			**uCA3**	uCA2	**uCA1**
		#1 (lateral digitation)		Deep hippocampal fissure	Dentate gyrus in the lateral hippocampal digitation (I)	CA3 in the lateral hippocampal digitation	CA2	CA1
	Prefimbrial hippocampus	#4 (medial digitation)	Vertical Hippocampus	Superficial hippocampal fissure	Upper/superior Band of Giacomini	**vCA3**	vCA2	**vCA1**
			Uncus	Superficial hippocampal fissure		**uCA3**	uCA2	**uCA1**
			Intralimbic gyrus	Superficial hippocampal fissure	Band of Giacomini	**uCA3, vCA3**		
		#2,3 (intermediate digitations)		Deep hippocampal fissure	Dentate gyrus in the intermediate hippocampal digitations (II, III)	CA3 in the medial hippocampal digitations	CA2, uCA2	CA1
		#1 (lateral digitation)		Deep hippocampal fissure	Dentate gyrus in the lateral hippocampal digitation (I)	CA3 in the lateral hippocampal digitation	CA2	CA1

Supplementary Table 9 Legend: Field distribution along the hippocampal longitudinal axis. Left columns include the different subregions of the human adult hippocampus along its longitudinal axis. Right columns include the different representatives of the hippocampal fissure and fields (hippocampal fissure → dentate gyrus → CA3 → CA2 → CA1). Bold indicates modified fields.
